# Management of patients with lower urinary tract symptoms due to benign prostatic enlargement at risk of progression treated with 5-alpha-reductase inhibitors: examining real-world clinical practice in Spain and Brazil

**DOI:** 10.1186/s12894-025-01966-6

**Published:** 2025-11-17

**Authors:** Juan Manuel Palacios, Pratiksha Kapse, Vanessa Cortés, Marcio Augusto Averbeck, Alberto Budia Alba, Danilo Souza Lima da Costa Cruz, Suryakant Somvanshi, Fiona Pereira

**Affiliations:** 1 Global Medical Urology, GSK, Madrid, Spain; 2 Global Medical Urology, GSK, Mumbai, India; 3 Global Medical Infectious Diseases, GSK, Bogota, Colombia; 4 Department of Urology, Moinhos de Vento Hospital, Porto Alegre, Brazil; 5 Department of Urology, Sao Lucas Hospital – PUCRS, Porto Alegre, Brazil; 6 Department of Urology, La Fe University and Polytechnic Hospital, Valencia, Spain; 7 Department of Urology, State University of Rio de Janeiro, Rio de Janeiro, Brazil; 8 Dev Biostats, India Stats, GSK, Bangalore, India; 9 Brand and Integrated Research Solutions, IQVIA, London, UK

**Keywords:** Benign prostatic enlargement, Lower urinary tract, 5-alpha-reductase inhibitors

## Abstract

**Background:**

For patients with lower urinary tract symptoms due to benign prostatic enlargement (LUTS/BPE), assessing patient risk of disease progression is important to determine if treatment with 5-alpha-reductase inhibitors (5ARIs) is warranted. Clinical trials and guidelines have identified risk factors for disease progression, which affects subsequent treatment decision making; however, real-world clinical practices may differ. This non-interventional, cross-sectional study examined the management of patients with LUTS/BPE at risk of disease progression receiving 5ARIs by urologists from Spain and Brazil.

**Methods:**

Urologists were randomly recruited and completed an online questionnaire comprising multiple-choice, open-ended ranking and rating questions on their clinical approach in patients with LUTS/BPE receiving 5ARIs. Urologists also provided patient record forms for two recent patients treated with 5ARIs.

**Results:**

In Spain and Brazil (*n* = 100 each), 66% and 75% of urologists, respectively, assessed patients for their risk of LUTS/BPE progression. The most frequent parameters used to assess risk of progression were: post-void residual volume (PVR: 79% Spain, 83% Brazil), prostate volume (PV: 78% Spain, 82% Brazil), and International Prostate Symptom Score (IPSS: 79% Spain, 79% Brazil). These were also the most frequent parameters considered before initiating 5ARIs: PVR (60% Spain, 78% Brazil); PV (87% Spain, 89% Brazil); and IPSS (66% Spain, 61% Brazil). Thresholds used to assess risk factors of disease progression were generally higher than recommended by clinical guidelines. Reasons for initiating 5ARIs included: to achieve sustained symptom relief (70% Spain, 71% Brazil) and reduce the risk of progression (63% Spain, 66% Brazil). Impact on sexual function was a barrier to 5ARI initiation (46% Spain, 67% Brazil).

**Conclusions:**

Most urologists in Spain and Brazil recognized the benefits of assessing the risk of disease progression in patients with LUTS/BPE. Many urologists reported assessing fewer risk factors and using higher thresholds than recommended by international guidelines, highlighting the need for ongoing medical education.

**Supplementary Information:**

The online version contains supplementary material available at 10.1186/s12894-025-01966-6.

## Introduction

 Benign prostatic enlargement (BPE) is a progressive condition characterized by reduced urine flow and lower urinary tract symptoms (LUTS) [1]. If left untreated or inappropriately treated, BPE can cause chronic high-pressure retention and long-term changes to bladder function [2–4]. Appropriate and timely treatment of BPE can reduce the risk of worsening LUTS, surgery and quality of life (QoL) deterioration [5, 6].

Treatment with 5alpha reductase inhibitors (5ARIs), as a monotherapy or in combination with alpha blockers, has been shown to improve clinical outcomes in patients with LUTS/BPE at risk of disease progression [7–9]. The American Urological Association (AUA) [5] clinical guidelines recommend that 5ARIs are used to treat patients with moderate-to-severe LUTS, while the European Association of Urology (EAU) [6] clinical guideline recommends that 5ARIs are used to treat men with moderate-to-severe LUTS and an increased risk of disease progression. Risk factors for disease progression can include: prostatic enlargement > 30–40 mL, prostate-specific antigen (PSA) > 1.5 ng/mL, advanced age, larger prostate volume (PV), larger post-void residual volume (PVR), and lower maximal urinary flow rate (Q_max_). However, some patients with risk factors for disease progression do not receive treatment with 5ARIs [10, 11].

The aim of this study was to assess the real-world management of patients with LUTS/BPE treated with 5ARIs by urologists in Spain and Brazil. Urologists responded to a questionnaire and completed patient record forms (PRFs). This study investigated the gap between guideline recommendations and real-world clinical practice to help optimize care for patients with LUTS/BPE at risk of progression.

## Methods

### Study design

**Fig. 1:**
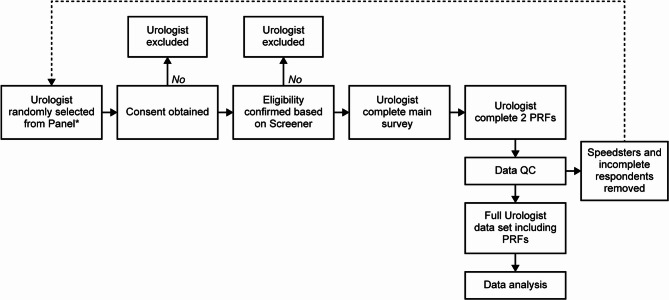
Study design. *Panel size in Spain was approximately 700 urologists; panel size in Brazil was approximately 1920 urologists. Urologists were randomly chosen from each panel and asked to participate in the questionnaire. To achieve the required sample size, 133 urologists were invited to participate in Spain of whom 105 met the inclusion criteria, and 153 were invited in Brazil, of whom 112 met the inclusion criteria. Urologists who did not complete the survey (5 in Spain and 12 in Brazil) were excluded from the final analysis resulting in 100 urologists in Spain and 100 in Brazil included in the final analysis. QC quality check, PRF patient record form

This non-interventional, cross-sectional study gathered data from urologists regarding the management of patients with LUTS/BPE from February–April 2023 (Fig. 1). Data were collected via an online survey comprising a questionnaire and PRFs. The questionnaire was designed to provide an overview of LUTS/BPE diagnosis, management, and monitoring. The PRFs were designed to collect information from each urologist about their two most recent patients with LUTS/BPE aged ≥ 50 who had initiated 5ARI therapy (alone or combination with an α-blocker) in the previous year.

### Respondents

Urologists were randomly recruited from Spain and Brazil using country-specific panels internal and external to the IQVIA network. IQVIA, formally known as the Intercontinental Medical statistics [IMS] Health and Quintiles network, is a global provider of advanced analytics, technology solutions, and clinical research services. Urologists provided consent to be contacted via email or telephone for research studies. The panels comprised approximately 700 urologists in Spain and 1920 in Brazil, from which eligible urologists were screened to achieve the target sample size. The survey questions used for screening are detailed in the Supplementary Methods, Additional file 1. Eligible urologists were practicing, with 5‒35 years of experience, spent ≥ 60% of their time in direct patient care, personally provided specialized assistance to ≥ 10 patients with LUTS/BPE in the month prior to the study, and were able to complete PRFs for the two most recent patients with LUTS/BPE aged ≥ 50 years who had initiated 5ARI therapy (alone or combination with an α-blocker) in the previous 12 months. Urologists were excluded if they reported that they had not treated an average of ≥ 10 different patients diagnosed with LUTS/BPE per month in the 12 months prior. Respondents were also excluded from the final analysis if they completed the survey in less than half the average time taken to do so across all respondents.

### Primary objective and endpoints

The primary objective of this study was to report real-world practice regarding the management of patients with LUTS/BPE at risk of progression receiving 5ARIs in Spain and Brazil. The key endpoints were to examine: (1) factors considered when assessing the risk of disease progression; (2) how the assessment of disease progression informs decisions about 5ARI treatment; (3) urologist adherence to guideline recommendations; (4) drivers of and barriers to 5ARI prescribing; and (5) patient involvement in treatment decisions. The survey questions used to assess all endpoints are detailed in the Supplementary Methods, Additional file 1.

### Ethical approval

This study complied with all applicable laws in the relevant countries regarding participant privacy. Urologists completed consent forms before study participation. All patient data were de-identified so informed consent was not required. A central independent review board (Pearl IRB) considered that the research had no risk or minimal risk to participants and was exempt from most of the requirements of the Federal Policy for the Protection of Human Subjects.

### Statistical analysis

A recruitment target of 100 urologists per country was selected based on feasibility assessments with advice from an expert on benign prostatic hyperplasia (BPH) with extensive experience on prevalence of LUTS/BPE and the patient population. To achieve the required sample size, 133 urologists were invited to participate in Spain, of whom 105 met the inclusion criteria, and 153 were invited in Brazil, of whom 112 met the inclusion criteria. Urologists who did not complete the survey (5 in Spain and 12 in Brazil) were excluded from the final analysis resulting in 100 urologists in Spain and 100 in Brazil included in the final analysis. Two PRFs were completed by each urologist, meaning that there were 200 patient cases per country and 400 patient cases reported-upon overall. The statistical analysis was primarily descriptive; frequencies and percentages of responses as well as mean and median thresholds are provided. The margin of error on the analysis performed was determined to be approximately 7% for the given sample size per country, with a 95% confidence interval. Therefore, sample size was considered adequate to support the quantitative analysis in this study. Pearson’s correlation test was used to evaluate the relationship between the proportion of patients at risk of progression and the proportion of patients taking 5ARIs. PASS software (NCSS, LLC, Version 22.0.2) was used for the analyses.

## Results

### Urologist characteristics and patient demographics

A total of 100 urologists were included from each country (Supplementary Table 1, Additional file 1). Urologists in Spain had an average of 16 years of clinical experience and those in Brazil had 14 years of clinical experience. Most urologists in Spain worked in public practice (87%) and most urologists in Brazil worked in private practice (65%). Across both countries, approximately half of the patients evaluated via PRFs were aged 55–64 years and diagnosed with LUTS/BPE > 1 year prior to study initiation. Most patients had multiple comorbidities, moderate LUTS, and were considered at risk of progression when they initiated 5ARI treatment (82% in Spain and 88% in Brazil; Supplementary Table 2, Additional file 1).

### Factors considered when assessing the risk of disease progression

Most urologists reported assessing patients with LUTS/BPE for their risk of disease progression (66% Spain; 75% Brazil, Table 1). The most common reason for not assessing progression in both countries was a lack of time during outpatient visits (55% of urologists in Spain and 52% in Brazil; Supplementary Table 3, Additional file 1).

**Table 1:** The proportion of patients diagnosed as at risk of progression and/or prescribed 5ARIs

Survey question		Spain	Brazil
S8	Patients with LUTS/BPE treated or managed in the last month, *n*	149	68
S9	Patients with LUTS/BPE currently being treated with 5ARIs, *n*	80	54
	Percentage of patients with LUTS/BPE treated or managed in the last month and currently being treated with 5ARIs, %	54	79
Q1a	Percentage of patients with LUTS/BPE assessed for risk of progression, %	66	75
Q2a	Percentage of patients with LUTS/BPE diagnosed as at risk of progression, %	39	42
Q10	Percentage of patients with LUTS/BPE prescribed 5ARIs, %	50	56
Q11	Percentage of patients with LUTS/BPE at risk of progression prescribed 5ARIs, %	65	81

*LUTS* lower urinary tract symptoms, *BPE* benign prostatic enlargement, *5ARI* 5-alpha-reductase inhibitor

In Spain, 39% of patients diagnosed with LUTS/BPE fulfilled progression criteria and were therefore identified as being at risk of progression (Table 1). From the questionnaires, the most common baseline parameters used to assess the risk of progression in Spain were symptom severity based on the International Prostate Symptom Score (IPSS; 79%), PVR (79%) and PV (78%) (Table 2). The mean thresholds used to decide if patients were at risk of progression for these factors were an IPSS of ≥ 17, PVR of ≥ 111 mL and PV of ≥ 60 mL. From the PRFs, these values were ≥ 20, ≥101 mL and ≥ 82 mL, respectively. PV assessments in Spain were conducted transabdominally (43%), via digital rectal examination (DRE; 32%) or transrectal ultrasound (22%). Baseline PSA levels were used to assess the risk of progression by 65% of urologists in Spain.

**Table 2:** Factors considered when assessing the risk of disease progression and the thresholds employed in Spain

	Questionnaires		PRFs			
	Q5a Parameters used to assess risk of progression (%) (*n* = 100)	Q5b Proportion of patients reported assessed with each parameter (%)*	Mean threshold (SD)	Median threshold (Max–Min)	Mean threshold (SD)	Median threshold (Max–Min)
Baseline symptom severity based on IPSS	79	59	17 (5.3)	18 (30–6)	20 (6.2)	20 (33–5)
Baseline PVR, mL	79	57	111 (37.3)	105 (200–25)	101 (42.0)	100 (200–1)
Baseline PV, mL	78	64	60 (31.7)	50 (153–17)	82 (28.6)	79 (165–17)
Baseline Q_max_, mL/sec	75	57	8 (2.7)	9 (12–3)	7 (2.2)	8 (13–2)
Baseline PSA, ng/mL	65	64	4 (1.8)	3 (8–2)	4 (1.8)	3 (9–1)
Symptom deterioration by clinical assessment	51	68	-	-	-	5 (8–1)
Baseline symptom severity based on clinical assessment	47	67	-	-	-	-
Age, years	47	65	67 (9.7)	67 (90–50)	-	-
Symptom deterioration while on alpha blocker monotherapy	41	57	5 (2.0)	5 (9–1)	5 (1.5)	5 (8–2)
Intravesical prostatic protrusion, mm	34	56	5 (1.9)	5 (10–2)	5 (1.8)	5 (8–1)
Renal function assessment (eGFR)	30	65	48 (12.6)	50 (77–22)	55 (15.9)	55 (90–29)
Bladder wall thickness measurement, mm	10	51	5 (1.7)	5 (8–2)	5 (2.3)	4 (10–1)
Frailty phenotype/status	9	44	-	-	-	-
Presence of metabolic syndrome	8	61	3 (0.8)	3 (4–2)	3 (0.8)	3 (4–2)
Evidence of chronic intraprostatic inflammation	7	72	-	-	-	-

*PRF* patient record form, *SD* standard deviation, *Max-Min* maximum, minimum, *IPSS* International Prostate Symptom Score, *PVR* post-void residual volume, *PV* prostate volume, *Q*_*max*_ maximal urinary flow rate, *PSA* prostate-specific antigen, *eGFR* estimated glomerular filtration rate

*Mean of reported percentage of patients from responses to the question “What percentage of your BPH patients do you use parameters listed below to assess risk of progression” (Q5A)

**Table 3:** Factors considered when assessing the risk of disease progression and the thresholds employed in Brazil

	Questionnaires		PRFs			
	Q5a Parameters used to assess risk of progression (%) (*n* = 100)	Q5b Proportion of patients reported assessed with each parameter (%)	Mean (SD)	Median (Max–Min)	Mean (SD)	Median (Max–Min)
Baseline PVR, mL	83	68	92 (33.7)	100 (180–30)	93 (37.8)	93 (200–13)
Baseline PV, mL	82	65	56 (18.5)	59 (116–29)	74 (25.2)	67 (180–33)
Baseline symptom severity based on IPSS	79	64	17 (5.2)	19 (30–4)	18 (5.3)	20 (31–7)
Age, years	74	66	61 (8.1)	60 (82–50)	-	-
Baseline symptom severity based on clinical assessment	64	74	-	-	-	-
Baseline Q_max_, mL/sec	56	46	8 (2.4)	9 (15–4)	8 (2.2)	8 (12–3)
Symptom deterioration while on alpha blocker monotherapy	51	65	5 (1.7)	5 (10–1)	5 (2.1)	5 (10–1)
Baseline PSA, ng/mL	49	65	3 (1.4)	3 (7–1)	4 (1.8)	3 (10–1)
Symptom deterioration by clinical assessment	48	71	-	-	-	-
Intravesical prostatic protrusion, mm	46	54	5 (2.6)	5 (10–1)	5 (2.7)	5 (10–1)
Bladder wall thickness measurement, mm	45	53	5 (2.2)	5 (10–1)	5 (1.8)	5 (10–1)
Renal function assessment (eGFR)	30	65	49 (13.2)	50 (80–21)	49 (15.5)	48 (90–15)
Presence of metabolic syndrome	19	40	3 (0.7)	3 (4–2)	3 (1.0)	3 (4–1)
Frailty phenotype/status	9	64	-	-	-	-
Evidence of chronic intraprostatic inflammation	7	44	-	-	-	-

*PRF* patient record form, *SD* standard deviation, *Max-Min* maximum/minimum, *PVR* post-void residual volume, *PV* prostate volume, *IPSS* International Prostate Symptom Score, *Q*_*max*_ maximal urinary flow rate, *PSA* prostate-specific antigen, *eGFR* estimated glomerular filtration rate

*Mean of reported percentage of patients from responses to the question “What percentage of your BPH patients do you use parameters listed below to assess risk of progression” (Q5A)

In Brazil, 42% of patients diagnosed with LUTS/BPE fulfilled progression criteria and were identified as being at risk of progression (Table 1). From the questionnaires, the most common baseline parameters used to assess the risk of progression in Brazil were PVR (83%), PV (82%) and symptom severity based on IPSS (79%; Table 3). The average thresholds used to decide if patients were at risk of progression for these factors were a PVR of ≥ 92 mL, a PV of ≥ 56 mL and an IPSS of ≥ 17. From the PRFs, these values were ≥ 93 mL, ≥ 74 mL and ≥ 18, respectively. PV assessments in Brazil were conducted transabdominally (44%), via DRE (32%) or transrectal ultrasound (10%).

Urologists from both countries most frequently reported that the long-term concerns of managing patients at risk of disease progression were acute and chronic urinary retention and QoL deterioration (Spain: 77%, 76% and 68% respectively; Brazil: 92%, 77% and 76%, respectively; Supplementary Fig. 1A–C, Additional file 1). Fewer noted bladder damage and remodeling as important long-term concerns, with 35% of respondents in Spain and 69% in Brazil rating bladder damage and remodeling as the most important long-term outcome. In the mid-term, urologists were most concerned about symptom deterioration (Spain: 87%, Brazil: 84%; Supplementary Fig. 1D, Additional file 1). When managing patients receiving α-blocker monotherapy, urologists were most often concerned about symptom progression (Spain: 92%, Brazil: 83%) and the risk of disease complications (Spain: 72%, Brazil: 90%; Supplementary Fig. 1E, Additional file 1).

### Assessment of diseas progression to infom decisions about 5ARI treatment

In our sample, the total proportion of patients with LUTS/BPE treated with 5ARIs (either alone or combination with an alpha-blocker) each month was 54% in Spain and 79% in Brazil (Table 1). Urologists reported prescribing 5ARIs to approximately half of all patients with LUTS/BPE (50% in Spain, 56% in Brazil) and to a higher proportion of patients diagnosed as being at risk of progression (65% in Spain and 81% in Brazil) via questionnaires.

From the questionnaires, the most common baseline parameters used to guide 5ARI treatment initiation were aligned with those used to assess the risk of progression (Tables 4 and 5) In Spain, the factors most frequently used to guide 5ARI treatment initiation were PV (87%), symptom severity per IPSS (66%) and PVR (60%). The mean thresholds for these factors used to drive 5ARI initiation were a PV of ≥ 59 mL, an IPSS of ≥ 17, and a PVR of ≥ 111 mL. From the PRFs, these values were ≥ 62 mL, ≥ 14 and ≥ 46 mL, respectively (Table 4).

**Table 4:** Factors considered when making decisions about 5ARI treatment and the thresholds employed in Spain

	Questionnaires	PRFs			
	Q9a Parameters used to decide risk on 5ARI treatment (%) (*n* = 100)	Mean (SD)	Median (Max-Min)	Mean (SD)	Median (Max-Min)
Baseline PV, mL	87	59 (26.4)	51 (147–22)	62 (24.4)	59 (137–28)
Baseline symptom severity based on IPSS	66	17 (5.6)	16 (27–4)	14 (6.1)	14 (25–2)
Baseline PVR, mL	60	111 (35.2)	103 (200–23)	46 (41.6)	32 (150–8)
Baseline PSA, ng/mL	59	3 (1.7)	2 (8–1)	3 (1.8)	2 (7–1)
Baseline symptom severity based on clinical assessment	58	-	-	-	-
Baseline Q_max_, mL/sec	56	8 (2.5)	9 (12–1)	10 (3.3)	10 (15–3)
Age, years	50	65 (9.6)	65 (86–50)		
Symptom deterioration while on alpha blocker monotherapy	50	5 (1.5)	5 (8–1)	4 (2.6)	4 (7–1)
Symptom deterioration by clinical assessment	43		-	-	-
Intravesical prostatic protrusion, mm	20	5 (1.9)	5 (10–2)	4 (4.1)	3 (10–1)
Renal function assessment (eGFR)	15	47 (13.5)	50 (62–18)	58 (21.1)	65 (90–20)
Bladder wall thickness measurement, mm	8	4 (1.9)	4 (6–1)	7 (4.2)	7 (10–4)
Evidence of chronic intraprostatic inflammation	8	-	-	-	-
Frailty phenotype/status	8	-	-	-	-
Presence of metabolic syndrome	5	2 (0.6)	2 (3–2)	4 (0.0)	4 (4–4)
Other	2		-	-	-

*PRF* patient record form, *5ARI* 5-alpha-reductase inhibitor, *SD* standard deviation, *Max-Min* maximum-minimum, *IPSS* International Prostate Symptom Score, *PV* prostate volume, *PVR* post-void residual volume, *PSA* prostate-specific antigen, *Q*_*max*_ maximal urinary flow rate, *eGFR* estimated glomerular filtration rate

**Table 5:** Factors considered when making decisions about 5ARI treatment and the thresholds employed in Brazil

	Questionnaires	PRFs			
	Q9a Parameters used to assess risk of progression (%) (*n* = 100)	Mean (SD)	Median (Max–Min)	Mean (SD)	Median (Max–Min)
Baseline PV, mL	89	56 (19.5)	50 (124–30)	56 (18.3)	53 (105–38)
Baseline PVR, mL	78	87 (32.4)	100 (153–30)	64 (33.2)	60 (120–10)
Baseline symptom severity based on clinical assessment	75	-	-	-	-
Baseline symptom severity based on IPSS	61	16 (4.8)	17 (24–7)	14 (4.6)	15 (20–7)
Age, years	56	62 (8.9)	60 (85–50)	-	-
Symptom deterioration while on alpha blocker monotherapy	59	5 (2.2)	5 (10–1)	4 (0.6)	4 (5–4)
Symptom deterioration by clinical assessment	50	-	-	-	-
Baseline Q_max_, mL/sec	49	8 (2.4)	8 (12–3)	8 (3.0)	9 (13–3)
Baseline PSA, ng/mL	35	3 (1.2)	4 (7–2)	3 (1.1)	3 (4–1)
Intravesical prostatic protrusion, mm	33	5 (2.4)	5 (10–1)	4 (1.2)	4 (5–3)
Bladder wall thickness measurement, mm	29	4 (1.9)	4 (10–2)	3 (1.2)	3 (6–2)
Renal function assessment (eGFR)	18	45 (17.1)	50 (70–15)	57 (21.9)	61 (90–23)
Evidence of chronic intraprostatic inflammation	8	-	-	-	-
Presence of metabolic syndrome	8	2 (0.5)	2 (3–2)	0 (0.0)	0 (0–0)
Frailty phenotype/status	5	-	-	-	-
Other	1	-	-	-	-

*PRF* patient record form; *SD* standard deviation; *Max-Min* maximum, minimum; *PV* prostate volume; *PVR* post-void residual volume; *IPSS* International Prostate Symptom Score; *Q*_*max*_ maximal urinary flow rate, *PSA* prostate-specific antigen; *eGFR* estimated glomerular filtration rate

In Brazil, these factors were PV (89%), symptom severity per IPSS (61%) and PVR (78%). The mean thresholds for these factors used to drive 5ARI initiation from the questionnaires were a PV of ≥ 56 mL, an IPSS of ≥ 16; and a PVR of ≥ 87 mL. From the PRFs, these values were ≥ 56 mL, ≥ 14 and ≥ 64 mL, respectively (Table 5).

### Urologist adherence to guideline recommendations

Most urologists in both Spain and Brazil (97% and 79%, respectively) reported following AUA and/or EAU guidelines in everyday practice (Supplementary Fig. 2, Additional file 1) and over half of urologists reported that guideline recommendations drove their decisions more than clinical knowledge (62% and 58%, respectively).

**Fig. 2:**
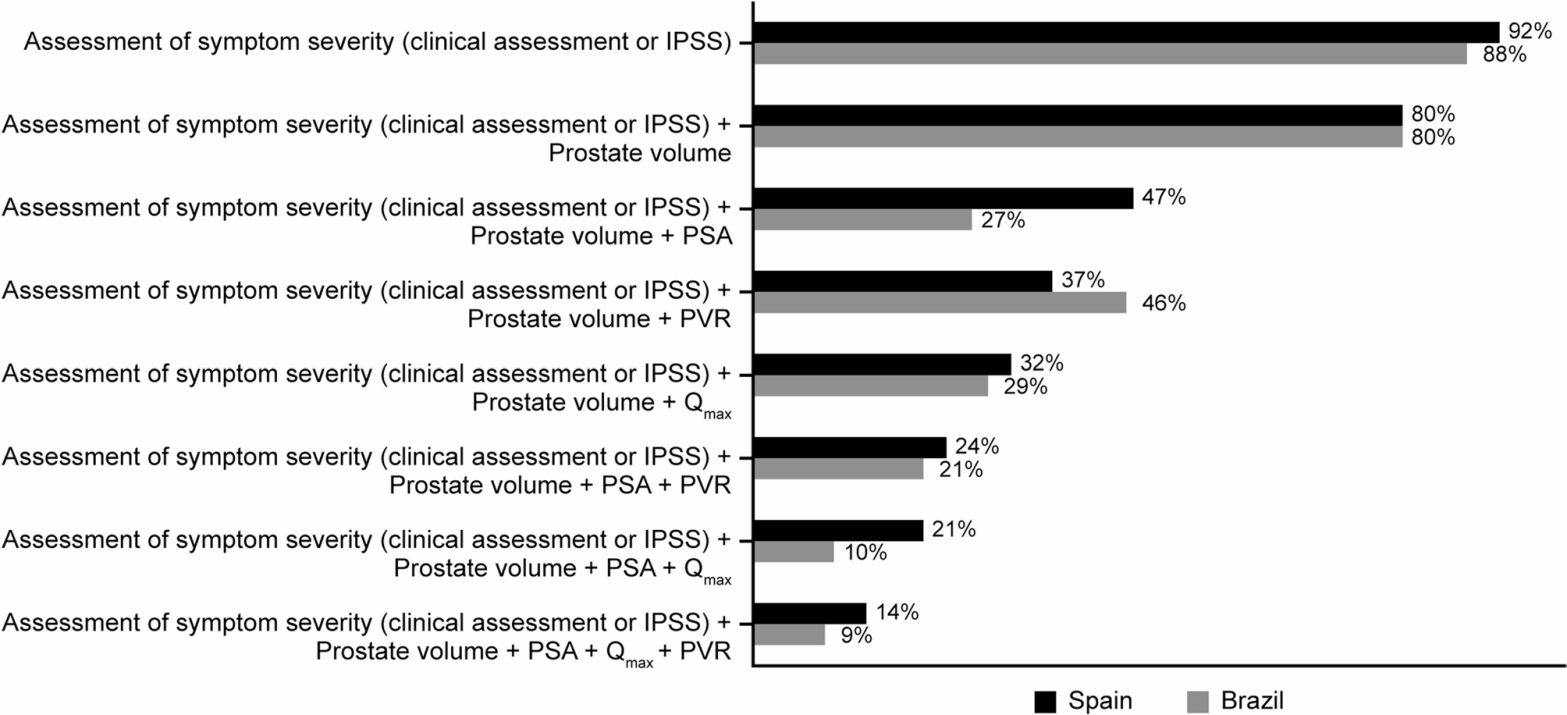
The individual and combined clinical parameters used by urologists to make 5ARI treatment decisions. 5ARI 5-alpha-reductase inhibitor, IPSS International Prostate Symptom Score, PSA prostate-specific antigen, PVR post-void residual volume, Q_max_ maximal urinary flow rate

From the questionnaires, fewer than half of urologists (47% in Spain and 27% in Brazil) reported using the guideline-recommended combination of IPSS, PSA and PV when making the decision to initiate 5ARIs (Fig. 2). In Spain and Brazil, respectively, the proportion of urologists who additionally included PVR in their assessment was 24% and 21%, and the proportion who additionally included Q_max_ was 21% and 10%. Only 14% and 9% of urologists in Spain and Brazil, respectively, used a combination of all 5 factors: IPSS, PV, PSA, PVR and Q_max_ (Fig. 2).

### Drivers of 5ARI intiation

**Fig. 3:**
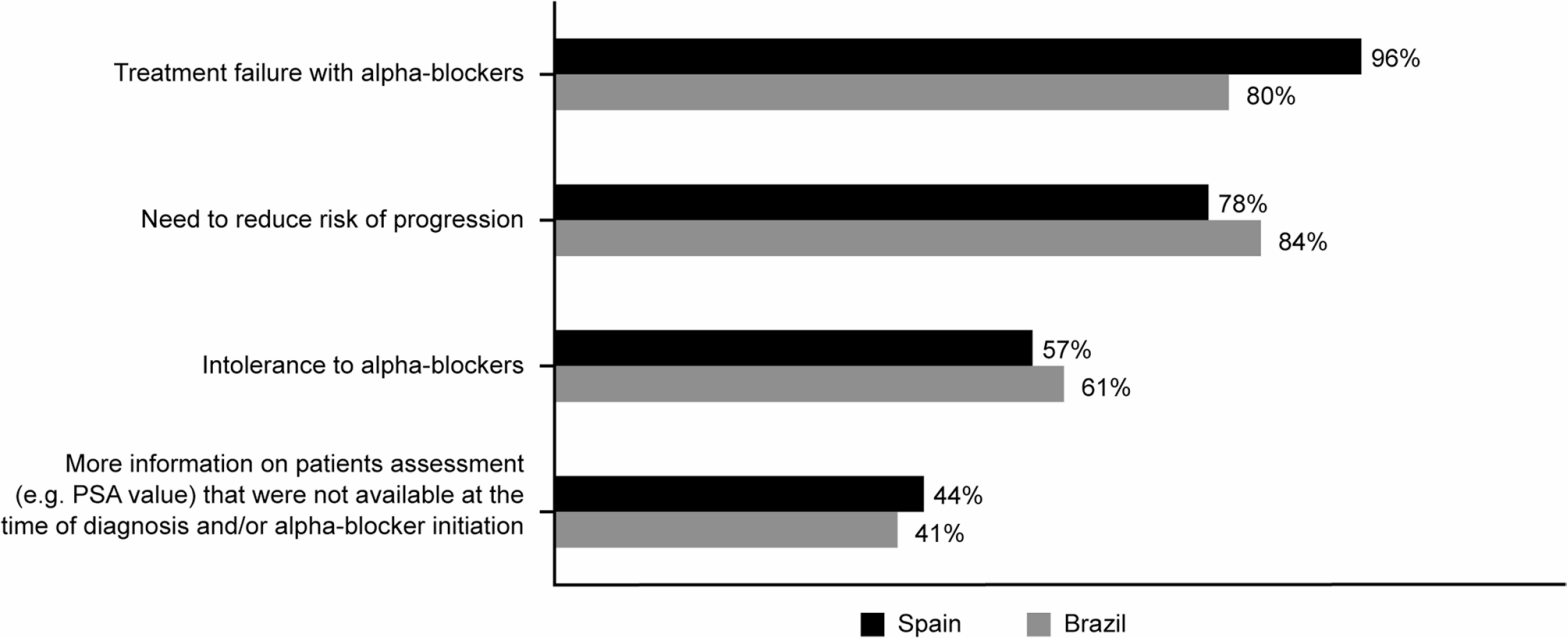
Factors considered when switching to 5ARI treatment per questionnaires. 5ARI 5-alpha-reductase inhibitor; PSA prostate-specific antigen

When selecting treatment, over 40% of urologists from both countries (44% in Spain and 46% in Brazil) reported in the questionnaires that reducing the risk of LUTS/BPE progression and reducing the risk of long-term complications were equally important considerations (Supplementary Fig. 3 A, Additional file 1). This was mirrored in the PRFs: the main reasons for initiating 5ARIs from the PRFs were aiming for sustained symptom relief (70% of urologists in Spain and 71% in Brazil) and a need to reduce the risk of progression (63% of urologists in Spain and 69% in Brazil; Table 6). From the questionnaires, the most common factor considered when switching to 5ARI therapy was treatment failure with α-blockers in Spain (reported by 96% of urologists) and a need to reduce the risk of progression in Brazil (84%; Fig. 3. The main urologist-reported benefit of 5ARI therapy was a reduced risk of LUTS/BPE-related surgery (84% of urologists in Spain and 59% in Brazil; Supplementary Fig. 3B, Additional file 1) and the main patient-perceived benefits reported by urologists were a better QoL (97% of urologists in Spain and 94% in Brazil) and sustained symptom relief (96% in Spain and 95% in Brazil; Supplementary Fig. 3 C, Additional file 1).

**Table 6:** Summary of concerns for progression, drivers for 5ARI initiation and barriers for 5ARI initiation. See supplement for full data

	Spain	Brazil
Concerns for progression most frequently rated ‘highly important’		
Acute urinary retention	77%	92%
Chronic urinary retention	76%	77%
Deterioration of quality of life	68%	76%
Chronic kidney failure	62%	73%
Most frequently reported drivers for 5ARI initiation		
Aiming for sustained symptom relief	70%	71%
Need to reduce risk of progression	63%	66%
Need to reduce risk of complications	33%	40%
High surgical risk/no surgical candidate	12%	10%
Patient expressed preference for a treatment that reduces disease complications	12%	9%
Intolerance to alpha blockers	12%	5%
Barriers to 5ARI initiation most frequently rated ‘highly important’		
When the patient is not keen to accept/trade any impact on sexual function	46%	67%
Preference to initiate alpha blockers as a first line	32%	45%
Delay 5ARIs to avoid any impact on sexual function	42%	43%
Patient is not suitable for long-term pharmacologic treatment	32%	32%
Concerns about patient affordability	4%	34%

*5ARI* 5-alpha-reductase inhibitor

### Barriers to 5ARI intiation

Almost half of the urologists in Spain (46%) and two thirds in Brazil (67%) reported patient’s unwillingness to accept any impact on sexual function as important to consider when thinking about 5ARI initiation in questionnaire responses (Supplementary Fig. 4A–B, Additional file 1). In Brazil, this view was reflected among 42% of urologists who wanted to delay 5ARI use to avoid an impact on sexual function, as reported in PRFs (Supplementary Fig. 4 C, Additional file 1). A preference to initiate α-blockers as first-line monotherapy was also reported as a key reason for not prescribing 5ARIs, as reported in PRFs (54% of urologists in Spain and 48% in Brazil).

### Patient involvement in treatment decision-making

Most urologists in Spain and Brazil (81% and 69%, respectively; Supplementary Fig. 5 A, Additional file 1) reported in questionnaire responses that they usually discuss therapy options with their patients; however, the final treatment decision is generally made by the urologist (54% of urologists in Spain and 66% in Brazil). Urologists in Spain reported in the questionnaire that 25% of discussions with patients ‘always’ led to a change in treatment, and 20% ‘often’ led to a change; these numbers were 20% and 22%, respectively, in Brazil (Supplementary Fig. 5B, Additional file 1). According to PRFs, most urologists discussed treatments with patients (88% in Spain and 93% in Brazil) and some of these discussions led to treatment changes (22% in Spain and 35% in Brazil; Supplementary Fig. 5 C, Additional file 1).

## Discussion

This study examined how urologists assess the risk of disease progression in patients with LUTS/BPE, and how that is used to inform 5ARI initiation in real-world clinical practice in Spain and Brazil. Urologists in both countries were generally aware of the need to monitor patients with LUTS/BPE for progression and recognized the benefits of 5ARI treatment. Although most urologists reported following AUA and/or EAU guidelines in everyday practice, we identified specific areas where assessment of the risk of disease progression could be improved, which could optimize access to 5ARI treatment by the right patient at the right time.

In this study, more than two thirds of urologists in both Spain and Brazil reported assessing the risk of disease progression in their patients with LUTS/BPE, with their main concerns being symptom deterioration and manifestations of bladder dysfunction (such as acute or chronic urinary retention). Fewer urologists reported bladder damage and remodeling as an important long-term consideration, which highlights the potential benefit of improved awareness of the consequences of chronic urinary obstruction [12].

Among urologists, the most common parameters used to assess the risk of disease progression were symptom severity (IPSS), PVR and PV, and urologists also widely used their subjective clinical assessment of symptoms. However, less than half of urologists reported using the combination of the three key parameters, IPSS, PSA and PV, recommended by AUA and EAU guidelines to assess the risk of disease progression and inform 5ARI initiation. Most urologists also did not combine these tests with additional factors like Q_max_ and PVR, to better inform the risk of progression. It is likely that this suboptimal approach to assessment of risk of disease progression in patients with LUTS/BPE is a direct consequence of the practical limitations of lack of time and availability of resources (e.g., flowmetry) experienced by healthcare systems.

Data from this study also shows that compared with treatment guidelines, urologists used higher thresholds for key parameters (e.g., PV, PVR) to identify patients at risk of disease progression and initiate treatment with 5ARIs. For example, the average baseline value of PV used to identify patients at risk of progression was 60 mL in Spain and 56 mL in Brazil, compared with the recommended thresholds of 30 mL per the AUA and 40 mL per the EAU guidelines [5, 6]. This difference in threshold between clinical practice and the guidelines was seen in both self-reported questionnaires and analysis of PRFs. It may be that urologists prescribe 5ARIs to patients with PV and PSA levels far above the thresholds recommended by the guidelines because the perceived benefit of treatment in these patients is greater. Potentially, this may mean that patients with relatively smaller prostates and lower PSA levels, who are nevertheless above the thresholds recommended by the guidelines and hence considered to be at risk of disease progression, do not receive 5ARI treatment from which they are likely to benefit. This is consistent with previous observations that patients with progression criteria were not receiving treatment with a 5ARI, either in monotherapy or in combination with an *α*-blocker, as is recommended by the clinical guidelines [10, 11]. Interestingly, the higher thresholds reported by the urologists in this study to initiate 5ARI treatment closely match the average profile of patients enrolled in landmark studies of 5ARI treatments, including the PLESS trial [7], the phase III trial of dutasteride monotherapy [13], the CombAT trial [8], and the CONDUCT trial [14]. In contrast, the thresholds recommended by the AUA and EAU guidelines reflect the entry criteria for these trials.

This observation reinforces the need for treatment guidelines to be supported by evidence of treatment outcomes at a more individualized patient level rather than by average risk profiles. This would help guide urologists on the relative importance of specific factors when considering 5ARIs and their combination with other therapies. A predictive modeling study in patients receiving either placebo, tamsulosin, dutasteride of a combination of tamsulosin and dutasteride for BPH, the severity of symptoms at baseline was found to be the most important predictor of treatment effect on IPSS change over time while PV, Q_max_ and PVR were most important when predicting the effect of treatment on the risk of acute urinary retention or BPE-related surgery [15]. A follow-up study noted that PVR, age and Q_max_ were predictors of changes in storage and voiding subscores [16].

This study provides useful insights into the factors that inform 5ARI therapy in patients with LUTS/BPE. Urologists in Spain and Brazil reported that the main barriers to starting 5ARI therapy were a reliance on *α*-blockers as first-line treatment, and the possible impact of 5ARIs on sexual function. These factors may be linked; previous studies have highlighted an impact of 5ARIs but not *α*-blockers on sexual function [17, 18]. Urologists main reason to switch to 5ARIs was failure to improve symptoms with *α*-blockers, reflecting evidence that 5ARIs IPSS, improve Q_max_ while decreasing PV and the risk of acute urinary retention and LUTS/BPE-related surgery [19].

In real-world practice, it is likely that urologists and primary care physicians will make treatment decisions holistically, considering both the benefit and the tolerability of the treatment rather than relying on IPSS, PVR and/or PV measurements as risk factors for disease progression. Further studies evaluating the impact of 5ARIs on patients with different levels of risk of progression would help physicians make informed treatment decisions by allowing them to consider the risks versus the benefits of 5ARIs. Additional patient factors (e.g., frailty and aging) and systemic factors (e.g., metabolic syndrome components and indicators of chronic inflammation) may also have an impact on disease progression and treatment decisions for people with LUTS/BPE [20–22]. Further research into these factors is required to fully understand how to optimize disease management.

The care of patients with LUTS/BPE could also be improved by optimizing shared decision-making practices; current guidelines emphasize the importance of patient preference regarding their treatments [23]. However, while most urologists in this study reported providing their patients with details on the therapy options available, few said that these conversations led to changes in treatment [23].

A strength of this study is the use of real-world, clinical practice data from Spain and Brazil, which supports the external validity in those countries. A limitation is that data were self-reported by urologists and may be prone to recall bias; however, analysis of PRFs helps mitigate this with objective data on the treatment decisions made in everyday clinical practice. Additionally, urologists were requested to complete PRFs for only the two most recent eligible patients. This approach may limit the representativeness of the sample, increase the risk of case-mix variability and may not fully capture the diversity of patients managed in routine practice. This study was also subject to selection bias and the sample included is likely not fully representative of urologists practicing in Spain and Brazil. However, these results provide insight into the real-world practice of urologists in two countries with high 5ARI use, which may help guide 5ARI prescribing, and PSA monitoring practices in countries where 5ARI use is less common.

## Conclusions

This study assessed the real-world management of patients with LUTS/BPE treated with 5ARIs from the perspective of urologists in Spain and Brazil. Most urologists in both countries were aware of the need to monitor patients with LUTS/BPE for disease progression and recognized the benefits of 5ARI treatment, especially in patients considered at risk of progression. Many urologists reported assessing fewer key risk factors and using higher thresholds than recommended by the guidelines, which may potentially limit the availability of 5ARI treatment for patients at risk of progression who may benefit from treatment. These results highlight the need for ongoing education and optimization of decision making for urologists treating patients with LUTS/BPE.

## Supplementary Information

Additional file 1: Management of patients with lower urinary tract symptoms due to benign prostatic enlargement at risk of progression treated with 5-alpha-reductase inhibitors: examining real-world clinical practice in Spain and Brazil

## Data Availability

The data that supports the findings of this study are available on request from the corresponding author. The data are not publicly available due to privacy or ethical restrictions.

## Abbreviations

5ARI: 5-alpha-reductase inhibitors
AUA: American Urological Association
BPE: benign prostatic enlargement
EAU: European Association of Urology
IPSS: International Prostate Symptom Score
LUTS: lower urinary tract symptoms
PRF: patient record form
PSA: prostate specific antigen
PV: prostate volume
PVR: post-void residual volume
Q_max_: post-void residual volume
QoL: quality of life

## References

[1] Kim EH, Brockman JA, Andriole GL. The use of 5-alpha reductase inhibitors in the treatment of benign prostatic hyperplasia. Asian J Urol. 2018;5:28–32. 10.1016/j.ajur.2017.11.005. 29379733 10.1016/j.ajur.2017.11.005 PMC5780290

[2] Ng M, Krishna MB, Baradhi KM. Benign Prostatic Hyperplasia. StatPearls. https://www.ncbi.nlm.nih.gov/books/NBK558920/. StatPearls. 2022. Accessed 23 Jul 2025. 32644346

[3] Bosch R, Abrams P, Averbeck MA, Finazzi Agró E, Gammie A, Marcelissen T, et al. Do functional changes occur in the bladder due to bladder outlet obstruction? - ICI-RS 2018. Neurourol Urodyn. 2019;38(Suppl 5):S56-65. 10.1002/nau.24076. 31278801 10.1002/nau.24076 PMC6915908

[4] Averbeck MA, De Lima NG, Motta GA, Beltrao LF, Abboud Filho NJ, Rigotti CP, et al. Collagen content in the bladder of men with LUTS undergoing open prostatectomy: a pilot study. Neurourol Urodyn. 2018;37:1088–94. 10.1002/nau.23418. 28945275 10.1002/nau.23418

[5] Lerner LB, McVary KT, Barry MJ, Bixler BR, Dahm P, Das AK, et al. Management of lower urinary tract symptoms attributed to benign prostatic hyperplasia: AUA guideline part I-initial work-up and medical management. J Urol. 2021;206:806–17. 10.1097/ju.0000000000002183. 34384237 10.1097/JU.0000000000002183

[6] European Association of Urology EAU Guidelines on non-neurogenic male lower urinary tract symptoms (LUTS). Arnhem. 2024; https://d56bochluxqnz.cloudfront.net/documents/full-guideline/EAU-Guidelines-on-Non-Neurogenic-Male-LUTS-2024.pdf. Accessed 11 Sept 2025.

[7] McConnell JD, Roehrborn CG, Bautista OM, Andriole GL Jr., Dixon CM, Kusek JW, et al. The long-term effect of doxazosin, finasteride, and combination therapy on the clinical progression of benign prostatic hyperplasia. N Engl J Med. 2003;349:2387–98. 10.1056/NEJMoa030656. 14681504 10.1056/NEJMoa030656

[8] Roehrborn CG, Siami P, Barkin J, Damião R, Major-Walker K, Nandy I, et al. The effects of combination therapy with dutasteride and Tamsulosin on clinical outcomes in men with symptomatic benign prostatic hyperplasia: 4-year results from the combat study. Eur Urol. 2010;57:123–31. 10.1016/j.eururo.2009.09.035. 19825505 10.1016/j.eururo.2009.09.035

[9] Crawford ED, Wilson SS, McConnell JD, Slawin KM, Lieber MC, Smith JA, et al. Baseline factors as predictors of clinical progression of benign prostatic hyperplasia in men treated with placebo. J Urol. 2006;175:1422–6. 10.1016/s0022-5347(05)00708-1. discussion 6–7. 16516013 10.1016/S0022-5347(05)00708-1

[10] Miñana B, Molero JM, Agra Rolán A, Martínez-Fornes MT, Cuervo Pinto R, Lorite Mingot D, et al. Real-world therapeutic management and evolution of patients with benign prostatic hyperplasia in primary care and urology in Spain. Int J Clin Pract. 2021;75:e14250. 10.1111/ijcp.14250. 33884719 10.1111/ijcp.14250 PMC8365648

[11] Bishr M, Boehm K, Trudeau V, Tian Z, Dell’Oglio P, Schiffmann J, et al. Medical management of benign prostatic hyperplasia: results from a population-based study. Can Urol Assoc J. 2016;10:55–9. 10.5489/cuaj.3058. 26977208 10.5489/cuaj.3058 PMC4771560

[12] Yaxley J, Yaxley W. Obstructive uropathy - acute and chronic medical management. World J Nephrol. 2023;12:1–9. 10.5527/wjn.v12.i1.1. 36704657 10.5527/wjn.v12.i1.1 PMC9846865

[13] Roehrborn CG, Boyle P, Nickel JC, Hoefner K, Andriole G. Efficacy and safety of a dual inhibitor of 5-alpha-reductase types 1 and 2 (dutasteride) in men with benign prostatic hyperplasia. Urology. 2002;60:434–41. 10.1016/s0090-4295(02)01905-2. 12350480 10.1016/s0090-4295(02)01905-2

[14] Roehrborn CG, Oyarzabal Perez I, Roos EP, Calomfirescu N, Brotherton B, Wang F, et al. Efficacy and safety of a fixed-dose combination of dutasteride and Tamsulosin treatment (Duodart^®^) compared with watchful waiting with initiation of Tamsulosin therapy if symptoms do not improve, both provided with lifestyle advice, in the management of treatment-naïve men with moderately symptomatic benign prostatic hyperplasia: 2-year CONDUCT study results. BJU Int. 2015;116:450–9. 10.1111/bju.13033. 25565364 10.1111/bju.13033

[15] Gravas S, Palacios-Moreno JM, Thompson D, Concas F, Kamola PJ, Roehrborn CG, et al. Understanding treatment response in individual profiles of men with prostatic enlargement at risk of progression. Eur Urol Focus. 2023;9:178–87. 10.1016/j.euf.2022.07.004. 35985933 10.1016/j.euf.2022.07.004

[16] Gravas S, Manuel-Palacios J, Chavan C, Roehrborn CG, Oelke M, Averbeck MA, et al. Modeling study of the effect of placebo and medical therapy on storage and voiding symptoms, nocturia, and quality of life in men with prostate enlargement at risk for progression. Prostate Cancer Prostatic Dis. 2024;27:469–77. 10.1038/s41391-023-00731-w. 37794168 10.1038/s41391-023-00731-w PMC11319195

[17] Corona G, Tirabassi G, Santi D, Maseroli E, Gacci M, Dicuio M, et al. Sexual dysfunction in subjects treated with inhibitors of 5α-reductase for benign prostatic hyperplasia: a comprehensive review and meta-analysis. Andrology. 2017;5:671–8. 10.1111/andr.12353. 28453908 10.1111/andr.12353

[18] Sokhal AK, Sankhwar S, Goel A, Singh K, Kumar M, Purkait B, et al. A prospective study to evaluate sexual dysfunction and enlargement of seminal vesicles in sexually active men treated for benign prostatic hyperplasia by alpha-blockers. Urology. 2018;118:92–7. 10.1016/j.urology.2017.08.025. 28860050 10.1016/j.urology.2017.08.025

[19] Kim JH, Baek MJ, Sun HY, Lee B, Li S, Khandwala Y, et al. Efficacy and safety of 5 alpha-reductase inhibitor monotherapy in patients with benign prostatic hyperplasia: a meta-analysis. PLoS ONE. 2018;13:e0203479. 10.1371/journal.pone.0203479. 30281615 10.1371/journal.pone.0203479 PMC6169865

[20] Bauer SR, Walter LC, Ensrud KE, Suskind AM, Newman JC, Ricke WA, et al. Assessment of frailty and association with progression of benign prostatic hyperplasia symptoms and serious adverse events among men using drug therapy. JAMA Netw Open. 2021;4:e2134427. 10.1001/jamanetworkopen.2021.34427. 34817584 10.1001/jamanetworkopen.2021.34427 PMC8613596

[21] Gandaglia G, Briganti A, Gontero P, Mondaini N, Novara G, Salonia A, et al. The role of chronic prostatic inflammation in the pathogenesis and progression of benign prostatic hyperplasia (BPH). BJU Int. 2013;112:432–41. 10.1111/bju.12118. 23650937 10.1111/bju.12118

[22] Sebastianelli A, Gacci M. Current status of the relationship between metabolic syndrome and lower urinary tract symptoms. Eur Urol Focus. 2018;4:25–7. 10.1016/j.euf.2018.03.007. 29602736 10.1016/j.euf.2018.03.007

[23] Emberton M. Medical treatment of benign prostatic hyperplasia: physician and patient preferences and satisfaction. Int J Clin Pract. 2010;64:1425–35. 10.1111/j.1742-1241.2010.02463.x. 20579137 10.1111/j.1742-1241.2010.02463.x

