# Supplementary material for: Management of patients with lower urinary tract symptoms due to benign prostatic enlargement at risk of progression treated with 5-alpha-reductase inhibitors: examining real-world clinical practice in Spain and Brazil
Source: BMC Urol. 2025 Nov 17;25:285. doi: 10.1186/s12894-025-01966-6 (PMC12625436; doi:10.1186/s12894-025-01966-6)
Supplement: Supplementary file 1 — Additional file 1: Management of patients with lower urinary tract symptoms due to benign prostatic enlargement at risk of progression treated with 5-alpha-reductase inhibitors: examining real-world clinical practice in Spain and Brazil [file 12894_2025_1966_MOESM1_ESM.docx]

**Supplementary Information for:**

Management of patients with lower urinary tract symptoms due to benign prostatic enlargement at risk of progression treated with 5-alpha-reductase inhibitors: examining real-world clinical practice in Spain and Brazil

**Supplementary Methods:** Screening questions and **r**elevant questions from the urologist survey.

| **SCREENING QUESTIONNAIRE** |
| --- |

| **S0** | **Programmer Note:**   - **SHOW ALL DEFINITIONS IN ONE SCREEN** - **SHOW THE SCREEN FOR AT LEAST 15 SECONDS**   **Benign Prostatic Hyperplasia (BPH)** is a progressive, non-malignant overgrowth of the prostate gland and the most common cause of lower urinary tract symptoms (LUTS) in ageing men.  **Please answer the following questions for your patients >=18 years** |
| --- | --- |

**S1 [SINGLE SELECT]**

Please select your Country

| **Country** | **Condition** |
| --- | --- |
| Spain | 1**[CONTINUE]** |
| Other | 99**[TERMINATE IMMEDIATELY]** |

**S2 [SINGLE SELECT]**

Please select your Primary medical specialty

| **Country** | **Condition** |
| --- | --- |
| Urologist | 1**[CONTINUE]** |
| General Physician | 2 **[TERMINATE IMMEDIATELY]** |
| Internal Medicine | 3 **[TERMINATE IMMEDIATELY]** |
| General Surgeon | 4 **[TERMINATE IMMEDIATELY]** |
| Other | 99**[TERMINATE IMMEDIATELY]** |

**S3 [SINGLE SELECT]**

Are you or someone in your immediate family, currently employed and / or paid by a pharmaceutical, biopharmaceutical, company, or healthcare manufacturer as a consultant or researcher (other than participating in clinical trials)?

|  | **Code** |
| --- | --- |
| Yes | 1 **[TERMINATE IMMEDIATELY]** |
| No | 2 **CONTINUE** |

**S4 [NUMERIC, RANGE 0% - 100%]**

What proportion of your professional time devoted to seeing and treating patients is spent in **public versus private practice?**

| Code | Practice setting |  |
| --- | --- | --- |
| 1 | Public practice | ______ % |
| 2 | Private practice | ______ % |
|  | **SHOW TOTAL, SUM MUST BE 100%** TOTAL | 100% |

**If Respondents Codes>= 50% In 01, consider in Public practice**

**If Respondents Codes> 50% In 02, consider in Private practice**

**S5 [NUMERIC; RANGE 0% – 100%]**
What proportion of your professional time is spent in direct patient care (as opposed to academic or administrative tasks)?

|  |  |
| --- | --- |
| Time spent in direct patient care | ___________ % |

**[TERMINATE IF <60%]**

**S6 [NUMERIC; RANGE 0 – 100]**

How many years have you been practicing as a Urologist?

|  | **Range 0 -100** | **Condition** |
| --- | --- | --- |
| 1 | ________ Years | **Continue if >5 and =< 35, else terminate** |

**S7 [SINGLE SELECT]**

Are you personally involved in the management and treatment of patients clinically diagnosed with Benign Prostatic Hyperplasia (BPH)?

|  |  | **Condition** |
| --- | --- | --- |
| 1 | Yes | **CONTINUE** |
| 2 | No | **TERMINATE** |
| 3 | Don’t Know | **TERMINATE** |

**S8 [NUMERIC; RANGE 0 – 999]**

Approximately, how many different patients diagnosed with Benign Prostatic Hyperplasia patients have you managed or treated personally in the last month? Please count the number of individual patients, not the number of visits.

|  |  | **Range 0 -999** |
| --- | --- | --- |
| 1 | **BPH patients managed or treated in the last month** | ________ patients |

**[TERMINATE IF S8 <10]**

**S9 [NUMERIC; RANGE 0 – 999]**

Of the **[INSERT S8 CODE 1]** BPH patients whom you have managed or treated in the last month; how many patients come under the following category:

|  |  | **Range 0 -999** |
| --- | --- | --- |
| 1 | **Currently in treatment for BPH with 5-ARIs in monotherapy or combination with an alpha-blocker** | ________ patients |

**[TERMINATE IF S9 <2]**

**S10 [SINGLE SELECT]**

Dear doctor, during this interview we would like to discuss with you the last **2 distinct cases of real patients**, that you actively managed with the following aspects:

- Aged 50 years or more
- Diagnosed with BPH
- Initiated on treatment with 5-ARIs (in monotherapy or combination with an alpha-blocker) by you in the last one year.

It is important that these are 2 distinct cases and are the most recent ones that you have consulted with prior to this interview. It goes without saying that we will respect the anonymity of these patients. In accordance with the code of conduct governing all market research, we will not ask you to reveal in any way or inform the identity of these patients, nor will we communicate the responses you provide in a form that would identify you as the respondent.

Could you please confirm that you can discuss these 2 cases of real patients? **(SINGLE ANSWER)**

|  | **Code** |
| --- | --- |
| No, I cannot discuss these 2 cases | 1 **TERMINATE** |
| Yes, I confirm that I can discuss these 2 cases | 2 **CONTINUE** |

**SCRIPT for terminated:**Thank you for your interest. We are sorry to inform you that you do not meeting all the criteria to participate in this study. We will have your answers in mind, and in case of any relevant changes in the recruitment criteria we will get in touch with you. We appreciate your collaboration, and hope that you will participate in future studies.

**SCRIPT for SCREEN-IN:**

Congratulations you have successfully qualified for this study and will now move to the main survey.

It will take around 30 minutes to complete. You have the right to withdraw at any time. Thank you for your time and participation.

The name of the sponsoring pharmaceutical company will be revealed to you at the end of the online survey, and you can change your mind about your consent at any time.

| **Questionnaire** |
| --- |

Q1. Of the BPH patients that you see per month, what percentage of BPH patients do you:

1. assess for risk of disease progression?
2. are diagnosed as “at risk of progression”?

Q2. For the BPH patients that you consider NOT requiring assessment for risk of progression, please select the main reasons for not doing so -

| **Reasons for not assessing risk of progression** |
| --- |
| Short time at outpatient practice |
| In my concept, risk of progression is not a decisive factor to choose treatment |
| I think risk of progression is overestimated in patients with BPH |
| I do not agree with clinical guidelines parameters and values that define risk of progression |
| I must follow institutional/hospital guidance for treating patients and risk of progression is not formal part of the assessment |
| My priority is to tackle symptoms, not risk of progression |
| Others (please specify) _______ |

Q3. What is the influence of international guidelines (EAU – AUA) recommended criteria vs. clinical knowledge (personal experience of managing BPH patients in the past) when assessing risk stratification in patients with BPH?

| **Impact on risk stratification** |
| --- |
| Guideline recommendations |
| Clinical knowledge (personal experience of managing BPH patients in the past) |

Q4. Which of the following guidelines do you follow for risk stratification of BPH patients?

| **Clinical guidelines followed** |
| --- |
| European guidelines - EAU Guidelines on Management of Non-Neurogenic Male Lower Urinary Tract Symptoms (LUTS), incl. Benign Prostatic Obstruction (BPO) |
| American guidelines - Management of Lower Urinary Tract Symptoms Attributed to  Benign Prostatic Hyperplasia: AUA GUIDELINE |
| Spanish Guidelines for LUTS/BPH |
| Brazilian Guidelines: LUTS/BPH |
| Other Local/Hospital guidelines/protocols |
| Others (please specify) _______ |
| None of the above |

Q5. Please select:

1. Based on your clinical experience, which of the following parameters do you use in your daily practice to assess risk of disease progression? Please select a minimum of 5 and maximum of
   10 parameters
2. What percentage of your BPH patients do you use parameters listed below to assess risk
   of progression?
3. For each parameter, please indicate the threshold value (such that if a patient’s test results are higher than this value), you consider the patient to be at increased risk of BPH progression

| **Parameter** |
| --- |
| Age |
| Baseline symptom’s severity based on IPSS |
| Baseline symptom severity based on clinical assessment |
| Baseline prostate volume |
| Baseline PSA |
| Baseline Q_max_ (uroflowmetry) |
| Baseline post-void residual volume (PVR) |
| Bladder wall thickness measurement |
| Renal function assessment (eGFR) |
| Symptom deterioration while on alpha blocker monotherapy |
| Symptoms deterioration by clinical assessment |
| Intravesical prostatic protrusion |
| Evidence of chronic intraprostatic inflammation |
| Frailty phenotype/status |
| Presence of metabolic syndrome |
| Others, please specify ( _______) |

| **Threshold that qualifies as increased risk of progression Q5c** | **Range of values** | | | | | | | |
| --- | --- | --- | --- | --- | --- | --- | --- | --- |
| Age | 50–100 years | | | | | | | |
| IPSS score | 0–35 points | | | | | | | |
| Symptom severity based on clinical assessment | Mild | Moderate | | | | | | Severe |
| Prostate volume | 0 mL – 180 mL | | | | | | | |
| PSA levels | 0 ng/mL – 10 ng/mL | | | | | | | |
| Uroflowmetry (Q_max_) | 0 mL/s to 15 mL/s | | | | | | | |
| Post-void residual volume (PVR) | 0 mL to 200 mL | | | | | | | |
| Bladder wall thickness measurement | 0 mm to 10 mm | | | | | | | |
| Renal function assessment (eGFR) | <15 to >90 mL/min | | | | | | | |
| Symptom deterioration while on alpha blocker monotherapy | 1 to more than 10 points | | | | | | | |
| Symptoms deterioration by clinical assessment | From mild to moderate | | | From moderate to severe | | | | |
| Intravesical prostatic protrusion | 1 mm to 10 mm | | | | | | | |
| Evidence of chronic intraprostatic inflammation | Yes | | | | | No | | |
| Approach for diagnosis | Histological finding in biopsy | Cytological parameters | | | | | Urinary and seminal plasma biomarkers (e.g., WBC count) | |
| Frailty phenotype/status | Yes | | | | No | | | |
| Approach for diagnosis | Clinical suspicion | Diagnosis by other specialty colleagues | | | | | Use of specific frailty index | |
| Presence of metabolic syndrome | 1 to 5 factors | | | | | | | |
| Approach for diagnosis | Clinical suspicion | | Diagnosis by other specialty colleagues | | | | | |
| Others, please specify | (Open text box) | | | | | | | |

Q6. Which of the following diagnostic tests do you use to assess Prostate volume in your BPH patients?

| **PV tests** |
| --- |
| DRE (digital rectal examination) |
| Transrectal ultrasound |
| Transabdominal US |
| CT scan |
| MRI |
| Others, please specify ( _______) |

Q7. Which of the following approaches do you follow when assessing symptom severity in your BPH patients?

| **Clinical symptom assessment** |
| --- |
| Self-filled IPSS by the patient |
| You or a nurse ask patients and fill IPSS |
| You ask patient about some key symptoms of the IPSS but do not do the whole IPSS |
| I conduct clinical evaluation based on my experience asking for symptoms without using IPSS |
| I do not evaluate symptom severity in BPH patients |
| Others, please specify ( _______) |

Q8. When thinking about treating BPH patients, please indicate your preference of attributes for a treatment for BPH on a scale of 1–7, where 1 indicates that reduction in risk of progression and long-term complications (*such as AUR-Surgery, UTI, incontinence, chronic kidney failure) is most important and 7 indicates that rapid symptom relief is most important

| **1**  **Reduction in risk of progression and long-term complications* is most important** | **2**  **Reduction in risk of progression and long-term complications* is significantly more important than symptom relief** | **3**  **Reduction in risk of progression and long-term complications* is slightly more important than symptom relief** | **4**  **Preference for reduction in risk o**f **progression and long-term complications = preference of symptom relief** | **5**  **Rapid symptom relief is slightly more important than reduction in risk of progression and long-term complications*** | **6**  **Rapid symptom relief is significantly more important than reduction in risk of progression and long-term complications*** | **7**  **Rapid symptom relief is most important** |
| --- | --- | --- | --- | --- | --- | --- |

Q9.

1. Which of the following clinical parameters are decisive to treat BPH patients with a 5ARI (in monotherapy or in combination with an alpha-blocker)? Please select a minimum of 5 and maximum of 10 parameters
2. Please select the importance (H/M/L) for each parameter when deciding to treat BPH patients with a 5ARI (in monotherapy or in combination with an alpha-blocker)
3. Please provide threshold for the parameters when deciding to treat BPH patients with a 5ARI (in monotherapy or in combination with an alpha-blocker)

| **Parameter** | **Use in deciding 5ARI prescription**  **Q9a** | **Importance of parameter (High/Medium/Low)**  **Q9b** |
| --- | --- | --- |
| Age |  |  |
| Baseline symptom’s severity based on IPSS |  |  |
| Baseline symptom severity based on clinical assessment |  |  |
| Baseline prostate volume |  |  |
| Baseline PSA |  |  |
| Baseline Q_max_ (uroflowmetry) |  |  |
| Baseline ost-void residual volume (PVR) |  |  |
| Bladder wall thickness measurement |  |  |
| Renal function assessment (eGFR) |  |  |
| Symptom deterioration while on alpha blocker monotherapy |  |  |
| Symptoms deterioration by clinical assessment |  |  |
| Intravesical prostatic protrusion |  |  |
| Evidence of chronic intraprostatic inflammation |  |  |
| Frailty phenotype/status |  |  |
| Presence of metabolic syndrome |  |  |
| Others, please specify ( _______) |  |  |

| **Threshold o**f **the parameters when deciding to treat BPH patients with a 5ARI**  **Q9c** | **Range of values**  **(SHOW SLIDER DISPLAYING LOWEST AND HIGHEST VALUE ONLY)** | | | | |
| --- | --- | --- | --- | --- | --- |
| Age | 50–100 years | | | | |
| IPSS score | 0–35 points | | | | |
| Symptom severity based on clinical assessment | Mild | Moderate | | | Severe |
| Prostate volume | 0 mL – 180 mL | | | | |
| PSA levels | 0 ng/mL – 10 ng/mL | | | | |
| Uroflowmetry (Q_max_) | 0 mL/s to 15 mL/s | | | | |
| Post-void residual volume (PVR) | 0 mL to 200 mL | | | | |
| Bladder wall thickness measurement | 0 mm to 10 mm | | | | |
| Renal function assessment (eGFR) | <15 to >90 mL/min | | | | |
| Symptom deterioration while on alpha blocker monotherapy | 1 to more than 10 points | | | | |
| Symptoms deterioration by clinical assessment | From mild to moderate | | | From moderate to severe | |
| Intravesical prostatic protrusion | 1 mm to 10 mm | | | | |
| Evidence of chronic intraprostatic inflammation | Yes | | | No | |
| Approach for diagnosis | Histological finding in biopsy | Cytological parameters | | | Urinary and seminal plasma biomarkers (e.g., WBC count) |
| Frailty phenotype/status | Yes | | | No | |
| Approach for diagnosis | Clinical suspicion | Diagnosis by other specialty colleagues | | | Use of specific frailty index |
| Presence of metabolic syndrome | 1 to 5 factors | | | | |
| Approach for diagnosis | Clinical suspicion | | Diagnosis by other specialty colleagues | | |
| Others, please specify | (Open text box) | | | | |

Q10. Of the BPH patients that you see per month, what percentage of BPH patients do you treat with 5ARIs?

Q11. Of the BPH patients that you diagnosed as “at risk of progression”, what percentage do you treat
with 5ARIs?

Q12. Which of the following factors prevent you from initiating 5ARI treatment (in monotherapy or in combination with an alpha-blocker) in your BPH patients at risk of progression?

Please provide importance of each factor as High/Medium/Low to indicate your preference

| **Barriers for prescribing 5ARIs** | **HIGH** | **MED** | **LOW** |
| --- | --- | --- | --- |
| Preference for conservative measures (watchful waiting, behavioral and dietary modifications) |  |  |  |
| Preference to initiate treatment with alpha blockers monotherapy as a first line |  |  |  |
| Preference for therapies providing rapid symptom relief |  |  |  |
| Concerned about patient affordability |  |  |  |
| Patient expressing preference for alpha blockers over 5ARIs |  |  |  |
| Wait for patients to have bigger volume prostates |  |  |  |
| Wait for patients to have more severe symptoms |  |  |  |
| Delay 5-ARI usage to avoid possible impact on sexual function |  |  |  |
| Delay 5-ARI usage to avoid possible complications in PSA monitoring |  |  |  |
| To avoid any possible complications because of DHT deprivation |  |  |  |
| Please specify which potential complication of DHT deprivation (open text) ___________ |  |  |  |
| Did not have diagnostic test results to conclude need for 5ARIs |  |  |  |
| When patient is not keen to accept/trade off any impact on sexual function |  |  |  |
| Patient is not suitable for long term pharmacological treatment |  |  |  |
| To reduce direct costs to patients or health system |  |  |  |
| Others (please specify) _______ |  |  |  |

Q13. Which of the following factors do you consider when switching a BPH patient at risk of progression from alpha blocker monotherapy to treatment with 5ARIs (in monotherapy or in combination with alpha blockers)?

| **Factors for switching** |
| --- |
| Treatment failure with alpha-blockers |
| Need to reduce risk of progression |
| More information on patients’ assessment (e.g., PSA value) that were not available at the time of diagnosis and/or alpha-blocker initiation |
| Intolerance to alpha- blockers |
| Others (please specify) _______ |

Q14. You mentioned that you consider treatment failure with alpha-blockers as reason to switch to 5ARIs (in monotherapy or in combination with alpha blockers)? In this context, how do you define treatment failure?

| **Definition of treatment failure with alpha blockers** |
| --- |
| Subjective lack of improvement referred by patient |
| Objective worsening of >4 points in the IPSS |
| Objective worsening of Q_max_ or no improvement |
| Lack of tolerance to alpha-blockers |
| Patients suffering of a new onset complication e.g., AUR, recurrent UTI, hematuria |
| Others (please specify) _______ |

Q15 When you discuss pharmacological treatment options with your BPH patients, either at the time of initiating, adding, or modifying their treatment, can you please indicate percentage of BPH patients that fall into each of these categories

| **Patient inputs on treatment** |
| --- |
| % of BPH patients with whom I rarely discuss pharmacological treatment options since they have minimal understanding of drug treatments and its outcomes |
| % of BPH patients with whom I have had a detailed discussion on pharmacological options and let the patient decide on options that work best for them |
| % of BPH patients whom I have informed about pharmacological treatment options, but I take final decision on what to prescribe |

Q16. When you discussed pharmacological treatment options with patients, what percentage of patients does it lead to a change in final treatment decision among BPH patients at risk of progression

| **Impact of patient discussion on treatment decisions** |
| --- |
| Always |
| Often |
| Sometimes |
| Rarely |
| Never |

**Note**: questions 17–21 were not used to assess the objectives of the publication. See protocol for the full questionnaire.

Q22 Which of following clinical outcomes concern you the most about BPH disease progression in the long term (around 4 years*)?

Please provide rating for each clinical outcome as High/Medium/Low to indicate its importance to you.

* Duration of follow up in landmark clinical trials like MTOPS or CombAT was around 4 years

| **Clinical outcomes of risk of progression** |
| --- |
| Symptom deterioration > 4 IPSS points |
| Deterioration of storage symptoms |
| Deterioration of voiding symptoms |
| PV increase |
| Bladder wall damage/remodeling |
| Acute urinary retention |
| Chronic urinary retention |
| Urinary tract infection |
| Hematuria |
| Bladder stones |
| Chronic kidney failure |
| Incontinence |
| Deterioration of quality of life |
| Others (please specify) _______ |

Q23. Please rank the diagnostic tests you use to monitor BPH progression in order of importance.

Please rank up to the 8 most important tests starting with ‘1’ as ‘most important’, ‘2’ as ‘2nd most important’, and ‘3’ as ‘3rd most important

| **Diagnostic tests** |
| --- |
| IPSS evaluation |
| Clinical symptoms evaluation |
| Prostate volume measurement using DRE |
| Prostate volume measurement using TRUS |
| Prostate volume measurement using Abdominal Ultrasound |
| PSA testing |
| Uroflowmetry (Q_max_) |
| Post-void residual volume (PVR) |
| Bladder wall thickness measurement |
| Renal function assessment (eGFR) |
| Urinary analysis |
| Intravesical prostatic protrusion measurement |
| Metabolic syndrome assessment (by yourself or from other specialists) |
| Urinary and seminal plasma biomarkers (e.g., WBC count) for intraprostatic inflammation |
| Cytological parameters for intraprostatic inflammation |
| Formal assessment of frailty (by yourself or from other specialists) |
| Others (please specify) _______ |

Q24. How often do conduct these tests in your BPH patient at risk of progression? Every 3, 6, 12 months. Less frequently

| **Diagnostic tests frequency** |
| --- |
| IPSS evaluation |
| Clinical symptoms evaluation |
| Prostate volume measurement using DRE |
| Prostate volume measurement using TRUS |
| Prostate volume measurement using Abdominal Ultrasound |
| PSA testing |
| Uroflowmetry (Q_max_) |
| Post-void residual volume (PVR) |
| Bladder wall thickness measurement |
| Renal function assessment (eGFR) |
| Urinary analysis |
| Intravesical prostatic protrusion measurement |
| Metabolic syndrome assessment (by yourself or from other specialists) |
| Urinary and seminal plasma biomarkers (e.g., WBC count) for intraprostatic inflammation |
| Cytological parameters for intraprostatic inflammation |
| Formal assessment of frailty (by yourself or from other specialists) |
| Others |

Q25. Based on your clinical experience, what are long term (around 4 years*)

1. benefits you see/experience when treating BPH patients with 5ARIs (in monotherapy or in combination with alpha blockers)?
2. patients perceived benefits according to you when using 5ARIs (in monotherapy or in combination with alpha blockers) for the treatment of BPH?

* Duration of follow up in landmark clinical trials like MTOPS or CombAT was around 4 years

|  |
| --- |
| Sustained symptom relief |
| Reduction in prostate volume |
| Reduction in risk of BPH-related surgery |
| Reduction in the risk of symptomatic deterioration |
| Better QoL |
| Reduction in the risk of bladder changes/remodeling secondary to obstruction |
| Reduction in the risk of recurrent UTI |
| Reduction in the risk of AUR |
| Reduction in the risk of incontinence |
| Reduction in the risk of chronic kidney failure (obstructive) |
| Others (please specify) _______ |

Q26. Based on your clinical experience, what are long term (around 4 years*)

1. concerns you see/experience when treating BPH patients with 5ARIs (in monotherapy or in combination with alpha blockers)?
2. patients perceived concerns according to you when using 5ARIs (in monotherapy or in combination with alpha blockers) for the treatment of BPH?

* Duration of follow up in landmark clinical trials like MTOPS or CombAT was around 4 years

|  |
| --- |
| Lack of efficacy, i.e., bothersome symptoms not being resolved |
| Complexity in interpretation of PSA levels changes |
| Negative impact on sexual function |
| Risk of masking prostate cancer (increased risk) |
| Overall negative impact on tolerability |
| Adherence |
| Affordability |
| Others (please specify) _______ |

Q27. You mentioned that negative impact on sexual function is an important concern for you in long term treatment of BPH patients with 5ARIs (in monotherapy or in combination with alpha blockers). Can you please rank top concerns regarding negative impact on sexual function?

Please rank top 3 reasons with 1 being the most important, 2 being the second most important and 3 being the third most important reason

| **Sexual function concerns** |
| --- |
| Erectile disfunction |
| Ejaculatory disfunction |
| Negative impact on libido/sexual desire |
| Negative impact on global sexual function |
| Others (please specify) _______ |

Q28. In the long term (duration of follow up in landmark clinical trials like MTOPS or CombAT was around
4 years) what concerns, if any, do you have regarding your BPH patients at risk of progression who are currently being treated with alpha blockers monotherapy?

| **Concerns of alpha blocker monotherapy** |  |
| --- | --- |
| Symptom’s progression |  |
| Risk of disease complications (e.g., AUR/surgery, incontinence, UTI) |  |
| Potential suboptimal outcomes if taken to surgery |  |
| Bladder damage/remodeling |  |
| Renal function deterioration |  |
| Others (please specify) _______ |  |
| None of the above |  |
| **Patient Record Forms** | |

PRF1. What is the age of this patient?

| **Age** |
| --- |
| Between 50 to 54 years old |
| Between 55 to 59 years old |
| Between 60 to 64 years old |
| Between 65 to 69 years old |
| Between 70 to 74 years old |
| Between 75 to 79 years old |
| More than 80 years old |

PRF2. When was this patient first diagnosed with BPH?

| **Time since diagnosis** |
| --- |
| In the last 6 months |
| 7 to 12 months ago |
| More than 12 months ago |

PRF3. Which comorbidities are present in this patient?

| **Comorbidities** |
| --- |
| Hypertension |
| Diabetes |
| Cardiovascular disease |
| Sexual disfunction |
| Options if sexual dysfunction is selected:  Erectile disfunction  Ejaculatory disfunction  Negative impact on libido/sexual desire  Negative impact on global sexual function  Others (please specify)__________ |
| Neurological condition |
| Type of neurological condition ____________ |
| Overweight/Obesity |
| Dyslipidemia |
| Non prostatic malignancy |
| No comorbidities |
| Others (please specify) _______ |

PRF4. Based on the clinical chart of this patient, when it was decided to prescribe 5ARIs to this patient, would you classify this patient to be at a risk of BPH progression?

| **Risk of progression** |
| --- |
| Yes |
| No |

PRF5. Based on the clinical chart of this patient, when you classified this patient as AT RISK OF PROGRESSION, what criteria did you use to take this decision?

| **Criteria for risk stratification** |
| --- |
| Clinical guidelines parameters and thresholds to define risk of progression |
| More and/or different parameters and thresholds, beyond those included in guidelines |

PRF6. Based on the clinical chart of this patient, when this patient was initiated with 5ARIs, which of the following diagnostic tests were conducted?

| **Diagnostic tests** |
| --- |
| IPSS evaluation |
| Prostate volume measurement using DRE |
| Prostate volume measurement using TRUS |
| Prostate volume measurement using Abdominal Ultrasound |
| PSA testing |
| Uroflowmetry (Q_max_) |
| Post-void residual volume (PVR) |
| Bladder wall thickness measurement |
| Renal function assessment (eGFR) |
| Urinary analysis |
| Intravesical prostatic protrusion measurement |
| Metabolic syndrome assessment (by yourself or from other specialists) |
| Urinary and seminal plasma biomarkers (e.g., WBC count) for intraprostatic inflammation |
| Cytological parameters for intraprostatic inflammation |
| Formal assessment of frailty (by yourself or from other specialists) |
| Others (please specify) _______ |

PRF7.

1. Please select all the factors that made you think that this patient is AT RISK OF BPH PROGRESSION? Please select a minimum of 5 and maximum of 10 parameters
2. Among the factors you selected, please indicate its importance (High/Medium/Low) in deciding risk stratification for this patient
3. Based on the clinical chart for this patient, what were the diagnostic test results for each parameter that you selected?

| **Parameter** | **Use in deciding this patient is AT RISK OF PRGRESSION**  **P7a** | **Importance of parameter in deciding this patient is AT RISK OF PROGRESSION (High/**  **Medium/ Low)**  **P7b** |
| --- | --- | --- |
| Age |  |  |
| Baseline symptom’s severity based on IPSS |  |  |
| Baseline symptom severity based on clinical assessment |  |  |
| Baseline prostate volume, mL |  |  |
| Baseline PSA |  |  |
| Baseline Q_max_ (uroflowmetry) |  |  |
| Baseline post-void residual volume (PVR) |  |  |
| Bladder wall thickness measurement, mm |  |  |
| Renal function assessment (eGFR) |  |  |
| Symptom deterioration while on alpha blocker monotherapy |  |  |
| Symptoms deterioration by clinical assessment |  |  |
| Intravesical prostatic protrusion, mm |  |  |
| Evidence of chronic intraprostatic inflammation |  |  |
| Frailty phenotype/status |  |  |
| Presence of metabolic syndrome |  | _ |
| Others, please specify ( _______) |  |  |

| **Threshold that qualifies as increased risk  of progression**  **P7c** | **Range of values**  **(SHOW SLIDER DISPLAYING LOWEST AND HIGHEST VALUE ONLY)** | | | | |
| --- | --- | --- | --- | --- | --- |
| IPSS score | 0–35 points | | | | |
| Symptom severity based on clinical assessment | Mild | | Moderate | | Severe |
| Prostate volume | 0 mL – 180 mL | | | | |
| PSA levels | 0 ng/mL – 10 ng/mL | | | | |
| Uroflowmetry (Qmax) | 0 mL/s to 15 mL/s | | | | |
| Post-void residual volume (PVR) | 0 mL to 200 mL | | | | |
| Bladder wall thickness measurement | 0 mm to 10 mm | | | | |
| Renal function assessment (eGFR) | <15 to >90 mL/Min | | | | |
| Symptom deterioration while on alpha blocker monotherapy | 1 to more than10 points | | | | |
| Symptoms deterioration by clinical assessment | From mild to moderate | | From moderate to severe | | |
| Intravesical prostatic protrusion | 1 mm to 10 mm | | | | |
| Evidence of chronic intraprostatic inflammation | Yes | No | | | |
| Approach for diagnosis | Histological finding in biopsy | | Cytological parameters | Urinary and seminal plasma biomarkers (e.g., WBC count) | |
| Frailty phenotype/status | Yes | | No | | |
| Approach for diagnosis | Clinical suspicion | | Diagnosis by other specialty colleagues | | Use of specific frailty index |
| Presence of metabolic syndrome | 1 to 5 factors | | | | |
| Approach for diagnosis | Clinical suspicion | | Diagnosis by other specialty colleagues | | |
| Others, please specify | (Open text box) | | | | |

PRF8.

1. Please select all the factors that made you think that this patient is NOT AT RISK OF BPH progression? Please select a minimum of 5 and maximum of 10 parameters
2. Among the factors you selected, please indicate its importance (High/Medium/Low) in deciding this patient is NOT AT RISK OF BPH progression
3. Based on the clinical chart for this patient, what were the diagnostic test results for each parameter that you selected?

| **Parameter** | **Use in deciding this patient is not AT RISK OF PRGRESSION**  **P8a** | **Importance of parameter in deciding this patient is NOT AT RISK OF PROGRESSION (High/**  **Medium/ Low)**  **P8b** |
| --- | --- | --- |
| Age |  |  |
| Baseline symptom’s severity based on IPSS |  |  |
| Baseline symptom severity based on clinical assessment |  |  |
| Baseline prostate volume |  |  |
| Baseline PSA |  |  |
| Baseline Q_max_ (uroflowmetry) |  |  |
| Baseline post-void residual volume (PVR) |  |  |
| Bladder wall thickness measurement |  |  |
| Renal function assessment (eGFR) |  |  |
| Symptom deterioration while on alpha blocker monotherapy |  |  |
| Symptoms deterioration by clinical assessment |  |  |
| Intravesical prostatic protrusion |  |  |
| Evidence of chronic intraprostatic inflammation |  |  |
| Frailty phenotype/status |  | _ |
| Presence of metabolic syndrome |  |  |
| Others, please specify ( _______) |  |  |

| **Threshold that qualifies as NOT AT RISK  OF PROGRESSION**  **P8c** | **Range of values**  **(SHOW SLIDER DISPLAYING LOWEST AND HIGHEST VALUE ONLY)** | | | |
| --- | --- | --- | --- | --- |
| IPSS score | 0–35 points | | | |
| Symptom severity based on clinical assessment | Mild | Moderate | | Severe |
| Prostate volume | 0 mL – 180 mL | | | |
| PSA levels | 0 ng/mL – 10 ng/mL | | | |
| Uroflowmetry (Q_max_) | 0 mL/s to 15 mL/s | | | |
| Post-void residual volume (PVR) | 0 mL to 200 mL | | | |
| Bladder wall thickness measurement | 0 mm to 10 mm | | | |
| Renal function assessment (eGFR) | <15 to >90 mL/min | | | |
| Symptom deterioration while on alpha blocker monotherapy | 1 to more than10 points | | | |
| Symptoms deterioration by clinical assessment | From mild to moderate | | From moderate to severe | |
| Intravesical prostatic protrusion | 1 mm to 10 mm | | | |
| Evidence of chronic intraprostatic inflammation | Yes | | | No |
| Approach for diagnosis | Histological finding in biopsy | Cytological parameters | | Urinary and seminal plasma biomarkers (e.g. WBC count) |
| Frailty phenotype/status | Yes | No | | |
| Approach for diagnosis | Clinical suspicion | Diagnosis by other specialty colleagues | | Use of specific frailty index |
| Presence of metabolic syndrome | 1 to 5 factors | | | |
| Approach for diagnosis | Clinical suspicion | Diagnosis by other specialty colleagues | | |
| Others, please specify | (Open text box) | | | |

PRF9. Based on the clinical chart of this patient, when you decided to initiate 5ARIs (in monotherapy or in combination), how did you assess symptoms?

| **Clinical symptoms assessment** |
| --- |
| Self-filled IPSS by the patient |
| You or a nurse asked the patient and fill IPSS |
| You asked patient about some key symptoms of the IPSS but did not do the whole IPSS |
| I conducted clinical evaluation based on my experience asking for symptoms without using IPSS |
| I did not evaluate symptom severity in this patient |
| Others, please specify ( _______) |

PRF10. Based on the clinical chart of this patient, thinking back to the visit when you decided to initiate 5ARIs (in monotherapy or in combination) to this patient, - how would you classify severity of clinical symptoms?

| **Clinical symptoms severity** | **Mild** | **Moderate** | **Severe** |
| --- | --- | --- | --- |
| IPSS score |  |  |  |
| Clinical evaluation of symptoms |  |  |  |

PRF11. Based on the clinical chart of this patient, when you decided to initiate 5ARIs to this patient, - please rank top 3 reasons to decide treatment with 5ARI (alone or in combination)?

Please rank top 3 reasons with 1 being the most important, 2 being the second most important and
3 being the third most important reason

| **Reasons for 5ARIs** |
| --- |
| Aiming for sustained symptoms relieve |
| Need to reduce risk of progression |
| Intolerance to alpha-blockers |
| Need to reduce risk of complications |
| Patient expressed preference for a treatment that reduces disease complications |
| High surgical risk/no surgical candidate |
| Others (please specify) _______ |

PRF12. Based on the clinical chart of this patient, when you decided to initiate 5ARIs, what were reasons for not prescribing 5ARIs prior to this?

| **Barriers to 5ARI prescription** |
| --- |
| Preference for conservative measures (watchful waiting, behavioral and dietary modifications) |
| Preference to initiate treatment with alpha blockers monotherapy as a first line |
| Preference for therapies providing rapid symptom relief |
| Concerned about patient affordability |
| Patient expressing preference for alpha blockers over 5ARIs |
| Wait for patients to have bigger volume prostates |
| Wait for patients to have more severe symptoms |
| Delay 5-ARI usage to avoid possible impact on sexual function |
| Delay 5-ARI usage to avoid possible complications in PSA monitoring |
| To avoid any possible complications because of DHT deprivation |
| Please specify which potential complication of DHT deprivation___________ |
| Did not have diagnostic test results to conclude need for 5ARIs |
| Patient was not keen to accept/trade off any impact on sexual function |
| Patient was not suitable for long term pharmacological treatment |
| To reduce direct costs to patient or health system |
| Others (please specify) _______ |

PRF13. Thinking back to the visit when you decided to initiate 5ARIs to this patient,

1. did you discuss pharmacological treatment options and their outcomes with this patient?
2. did this lead to change in final treatment decision?

| **Patient discussion** | **Yes** | **No** |
| --- | --- | --- |
| Discussion with patient on pharmacological treatment options and their outcomes |  |  |
| Change in final treatment decision |  |  |

PRF14. How did you interpret PSA values in this patient?

| **PSA interpretation** |
| --- |
| Using the doubling rule |
| Evaluating any confirmed increase from nadir value while on 5ARI |
| Monitoring absolute (unadjusted) values |
| I did not monitor PSA levels in this patient |
| Others (please specify) _______ |

PRF14a. You mentioned that you use the doubling rule to interpret PSA level in this patient, can you please specify which option below best describes your approach with this patient

| **PSA interpretation** |
| --- |
| Correction of PSA values (doubling of the PSA value) after six months of therapy |
| Correction of PSA values (doubling of the PSA value) after 1 year of therapy |
| Others (please specify) _______ |

PRF15. What concerns you most in terms of disease progression for this patient in

1. mid-term (1 to 3 years)
2. long- term (3 to 5 years)

| **Concerns in mid and long term** | **Mid-term concerns**  **P13a** | **Long-term concerns**  **P13b** |
| --- | --- | --- |
| Symptom deterioration |  |  |
| PV increase |  |  |
| Bladder wall damage |  |  |
| Acute urinary retention |  |  |
| Chronic urinary retention |  |  |
| Hematuria |  |  |
| Urinary tract infection |  |  |
| Bladder stones |  |  |
| Renal dysfunction |  |  |
| Incontinence |  |  |
| Erectile dysfunction |  |  |
| Renal function deterioration |  |  |
| Others (please specify) _______ |  |  |

**Supplementary Figure 1**: Clinical concerns reported by urologists treating patients with LUTS/BPE at risk of progression: (a) long-term outcomes per questionnaires in Spain; (b) long-term outcomes per questionnaires in Brazil; (c) long-term outcomes per PRFs in both countries; (d) mid-term outcomes per PRFs in both countries; and (e) long-term outcomes for patients at risk of progression receiving α blocker monotherapy per PRFs in both countries.

a)


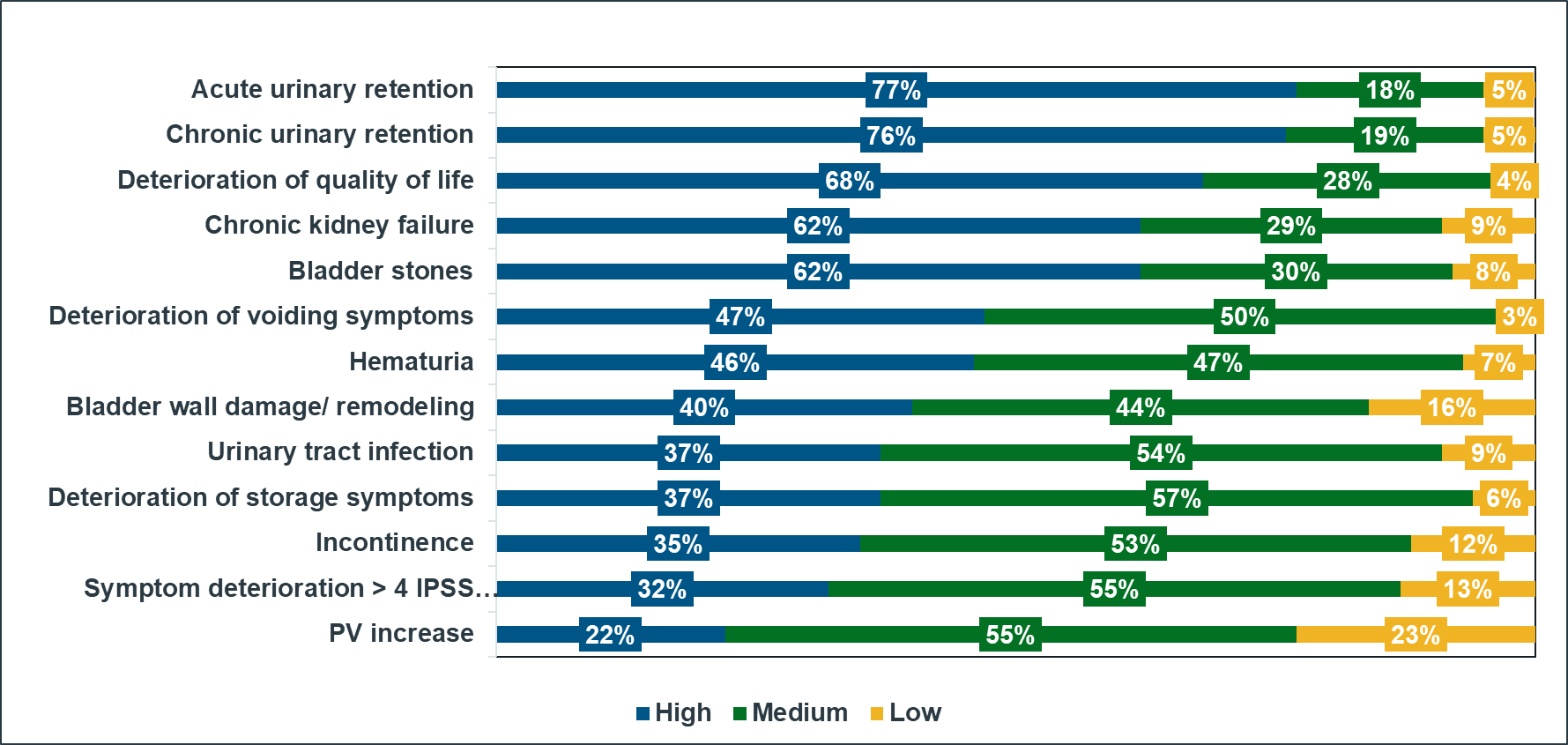


b)


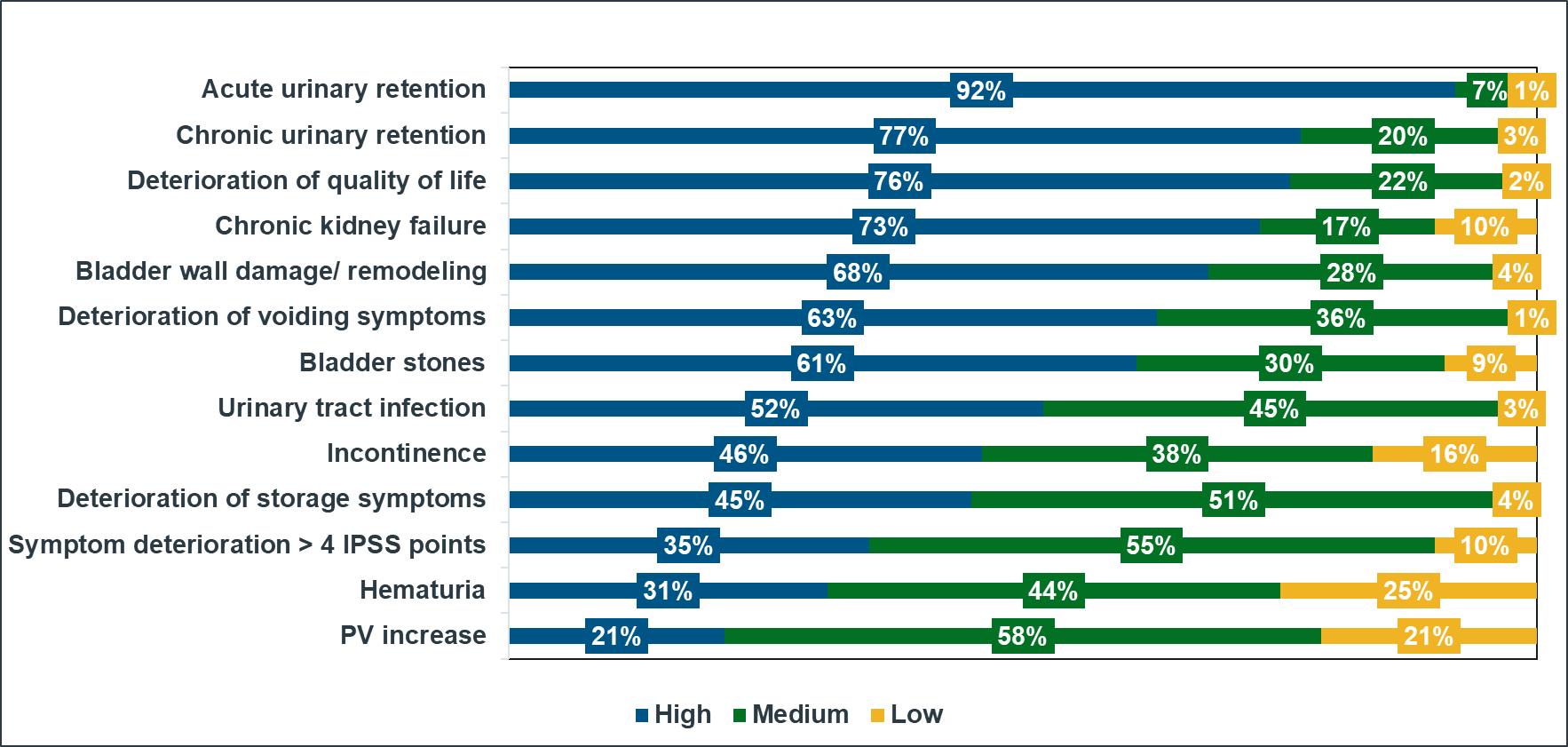


c)


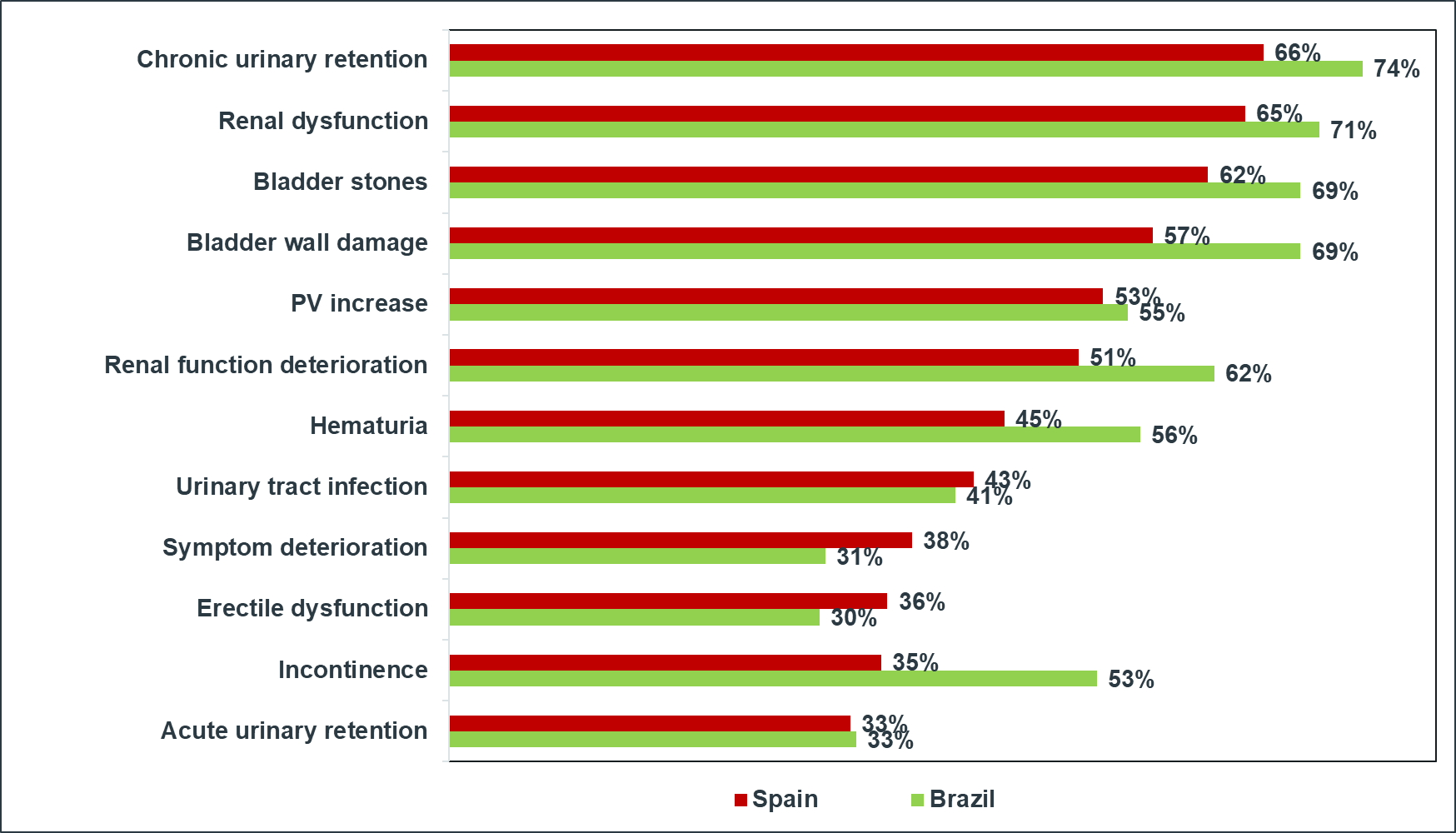


d)


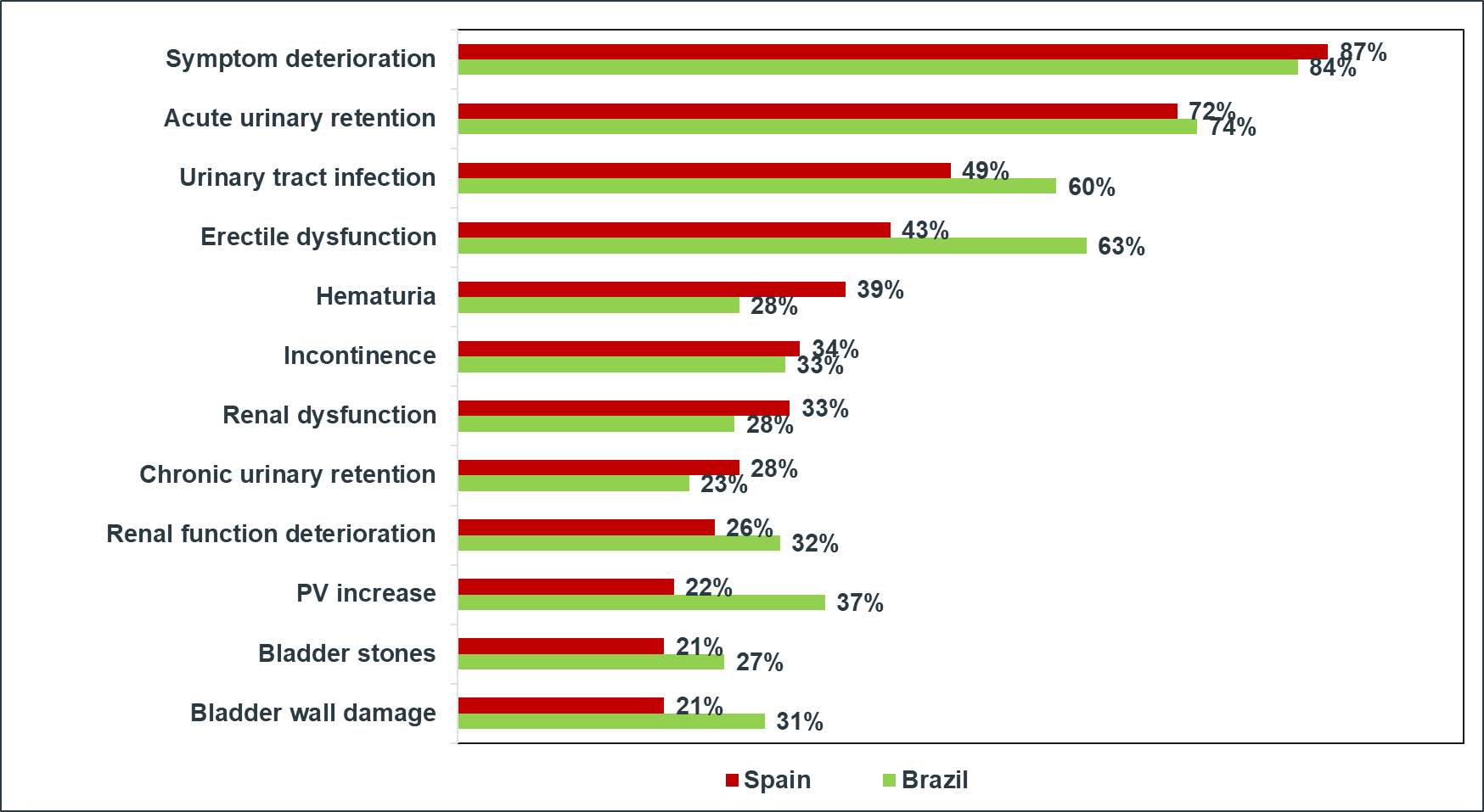


e)

***
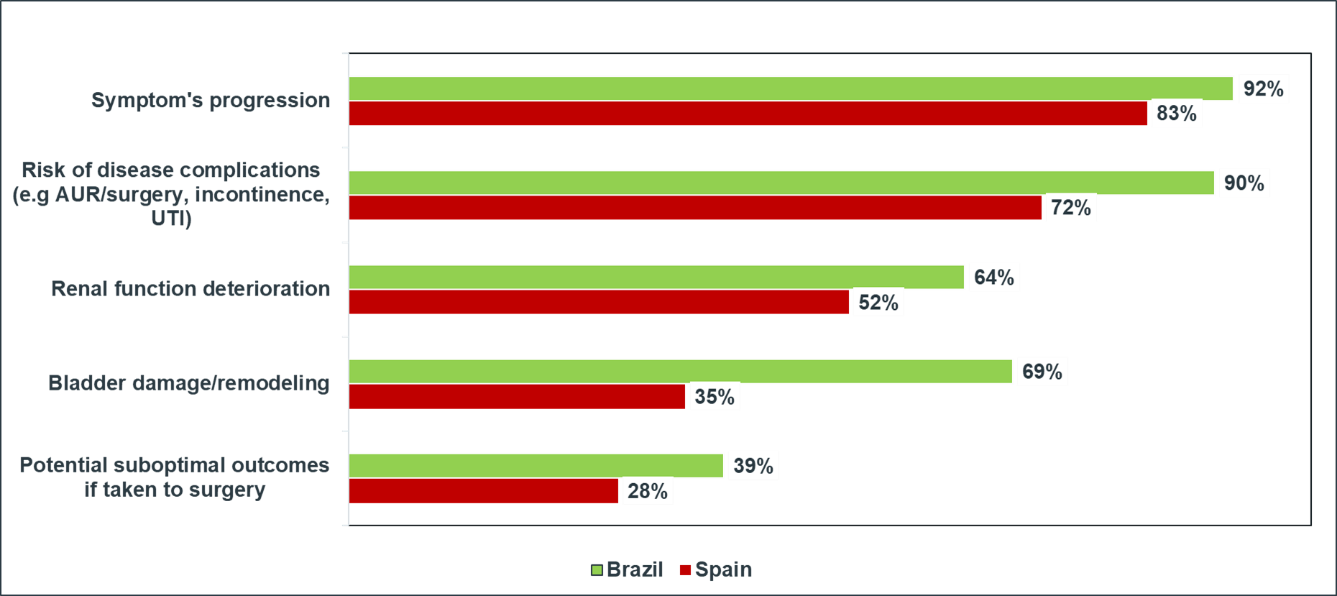
***

5ARI, 5-alpha-reductase inhibitor; α blocker, alpha-adrenergic antagonist; BPE, benign prostatic enlargement; IPSS, International Prostate Symptom Score; LUTS, lower urinary tract symptoms; PRF, patient record form; PV, prostate volume.

**Supplementary Figure 2:** Guidelines used by urologists when assessing the risk of LUTS/BPE progression per (a) questionnaire and (b) PRFs.

a)


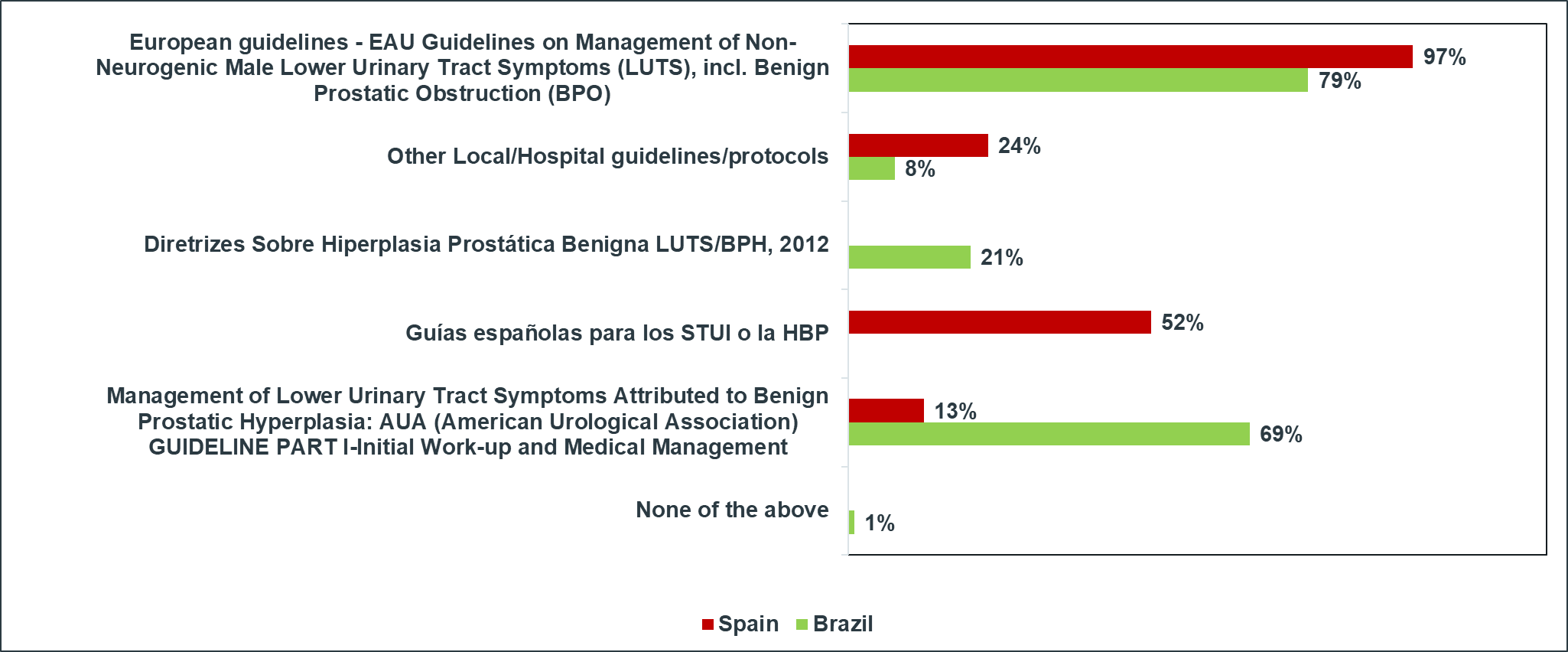


b)

***
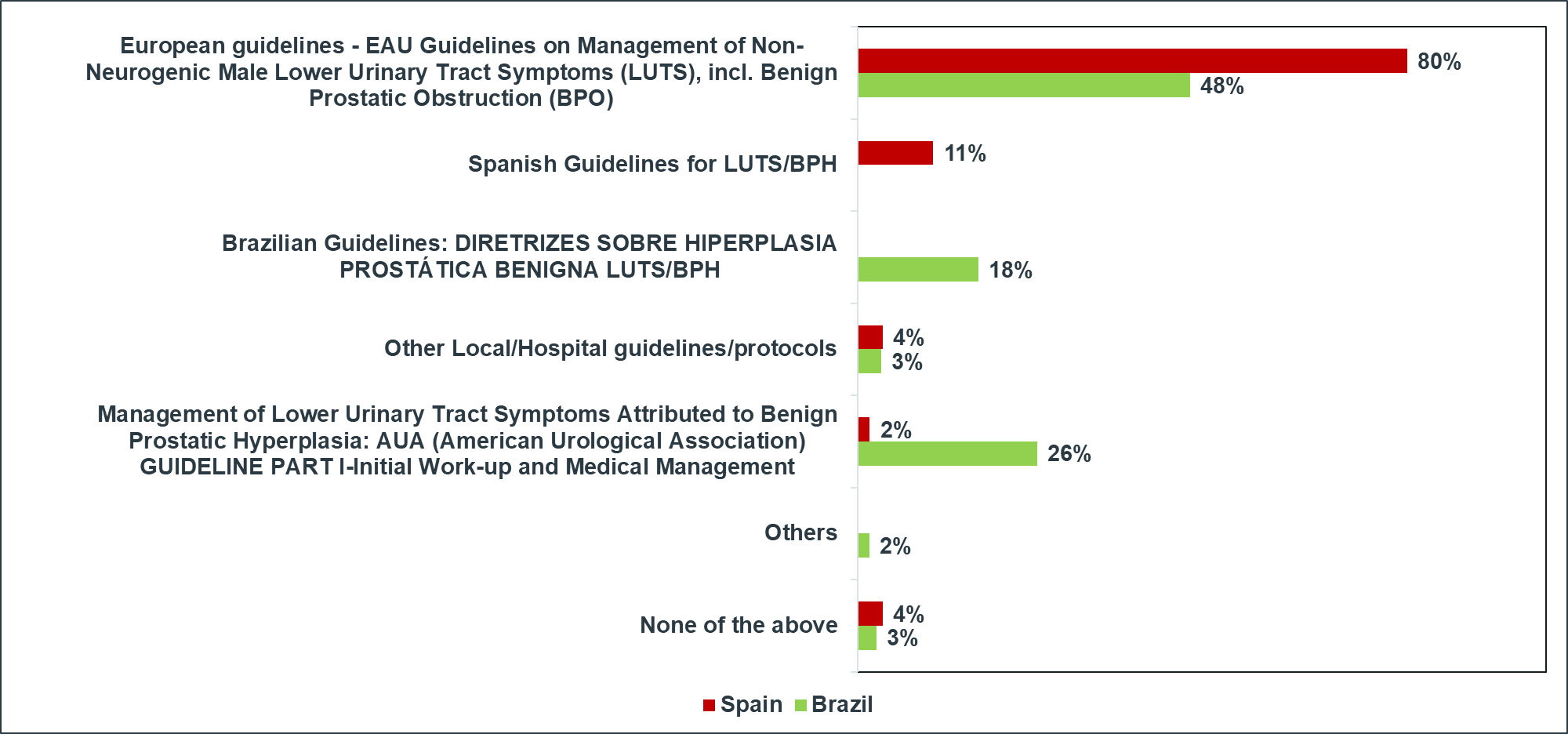
***

BPE, benign prostatic enlargement; BPO, benign prostatic obstruction; EAU, European Association of Urology; LUTS, lower urinary tract symptoms; PRF, patient record form.

**Supplementary Figure 3:** Drivers of 5ARI initiation: (a) the relative importance of symptom relief and long-term complications per questionnaires; (b) urologist-reported benefits of long-term 5ARI therapy per questionnaires; (c) urologist-reported patient-perceived benefits of long-term 5ARI therapy per questionnaires.

a)


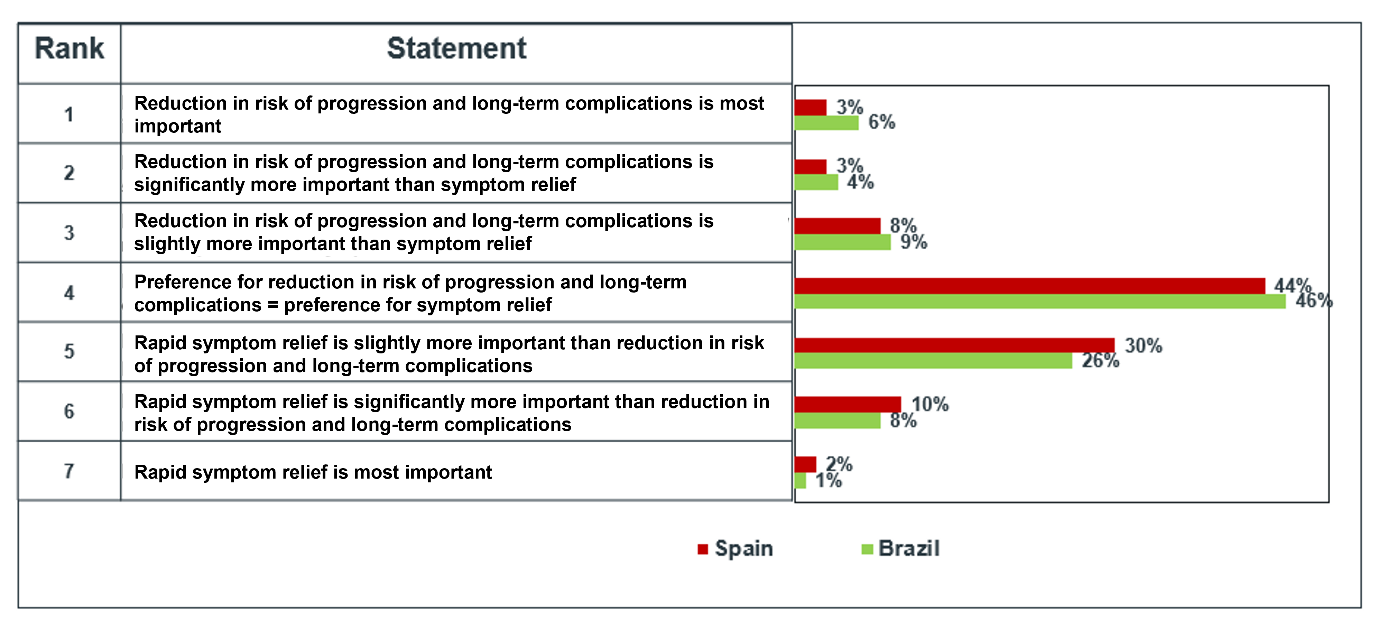


b)


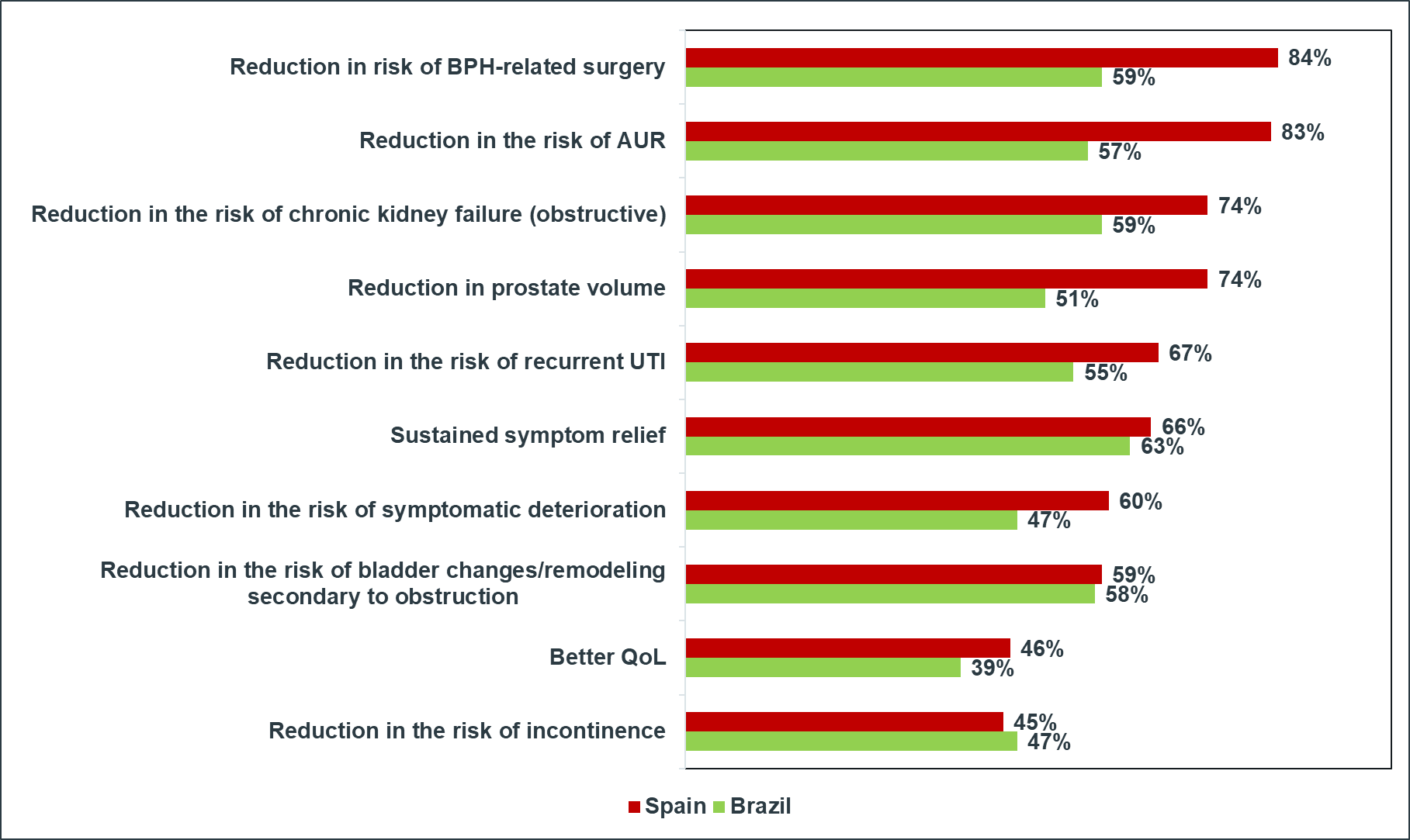


c)
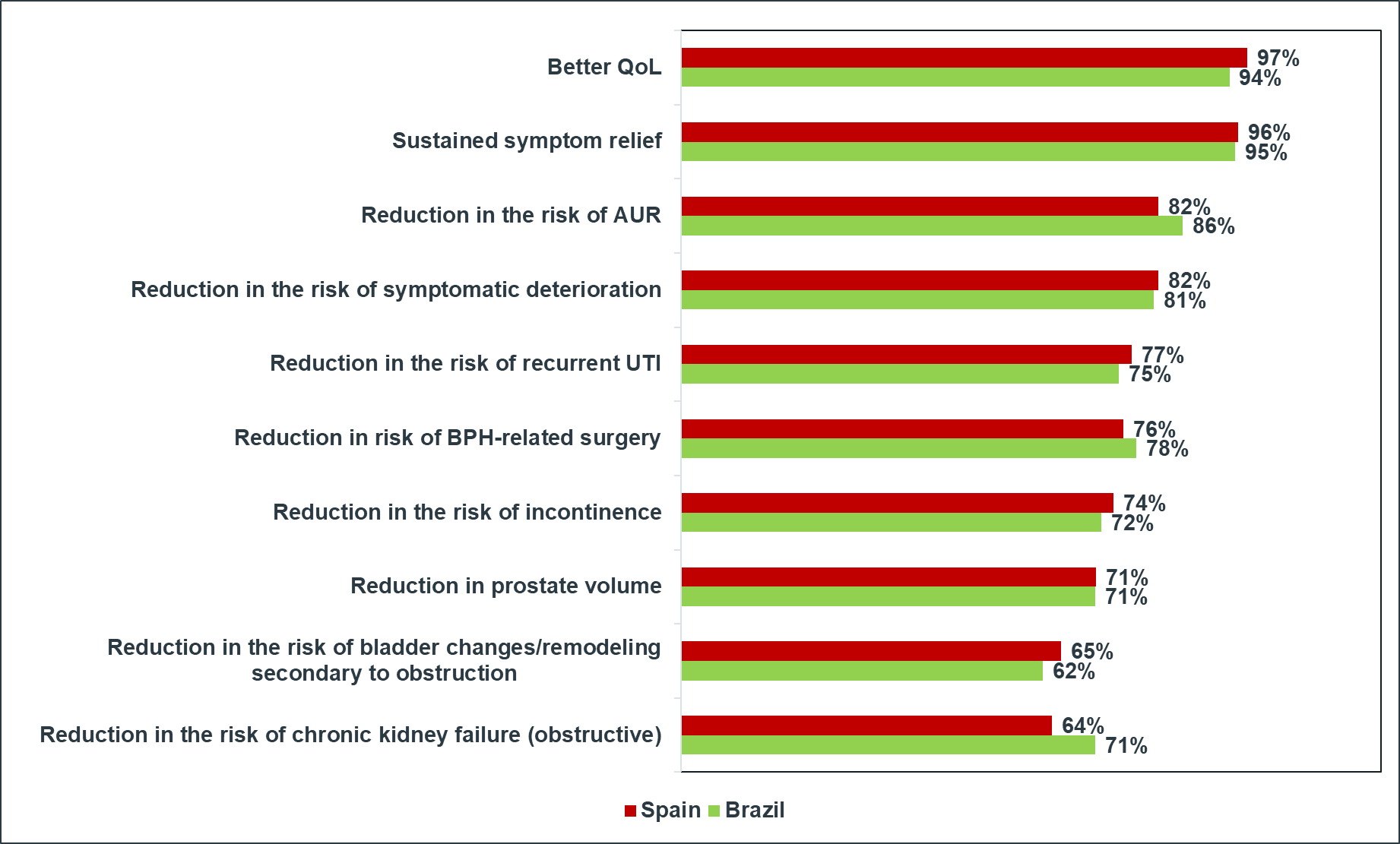


5ARI, 5-alpha-reductase inhibitor; BPH, benign prostatic hyperplasia; PRF, patient record form; PSA, prostate-specific antigen; QoL, quality of life; UTI, urinary tract infection.

**Supplementary Figure 4:** Barriers to 5ARI initiation: (a) the most important barriers in Spain per questionnaires; (b) the most important barriers in Brazil per questionnaires; and (c) the main reasons for not prescribing 5ARIs in both countries per PRFs.

a)

***
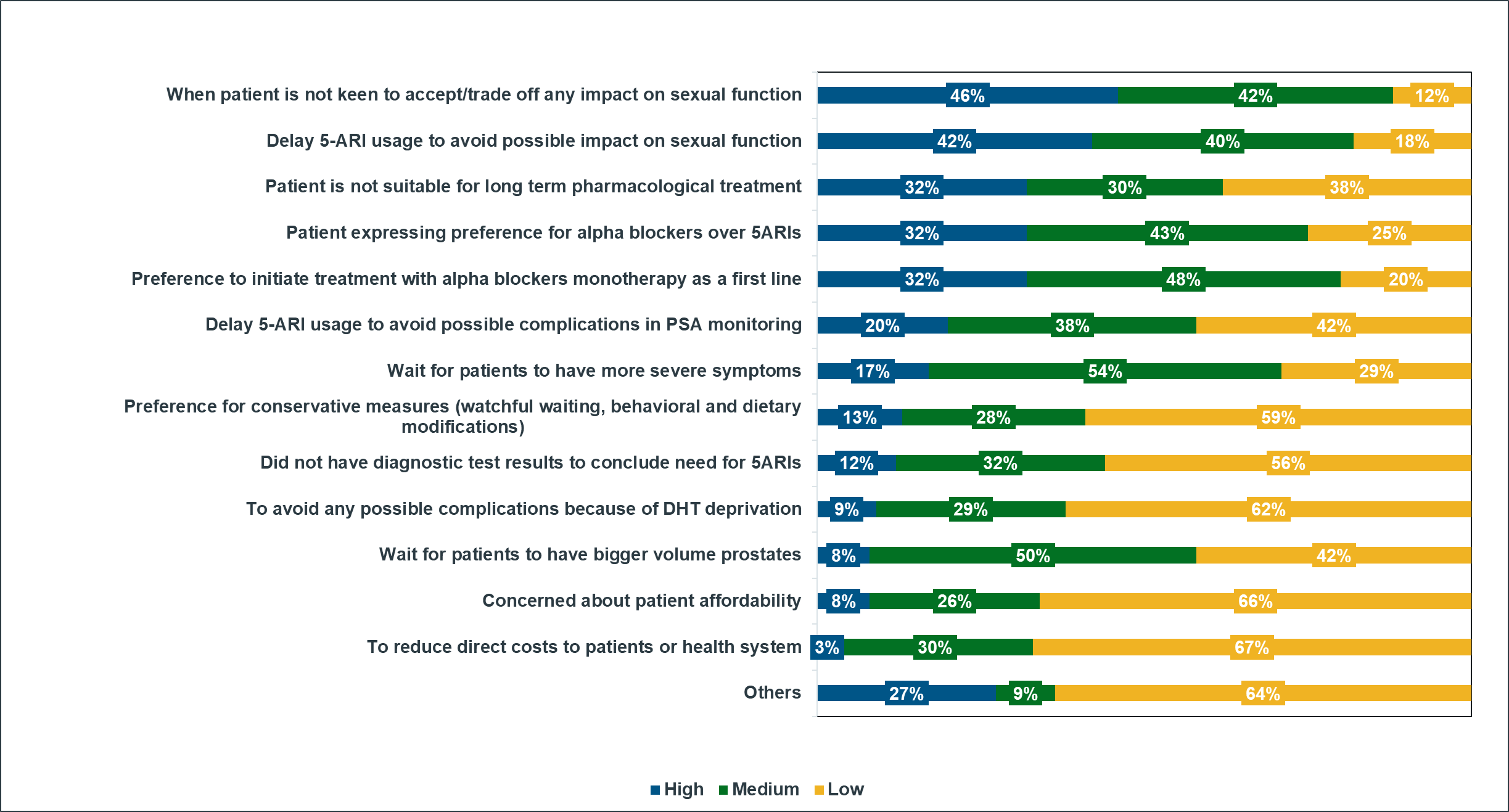
***

b)

***
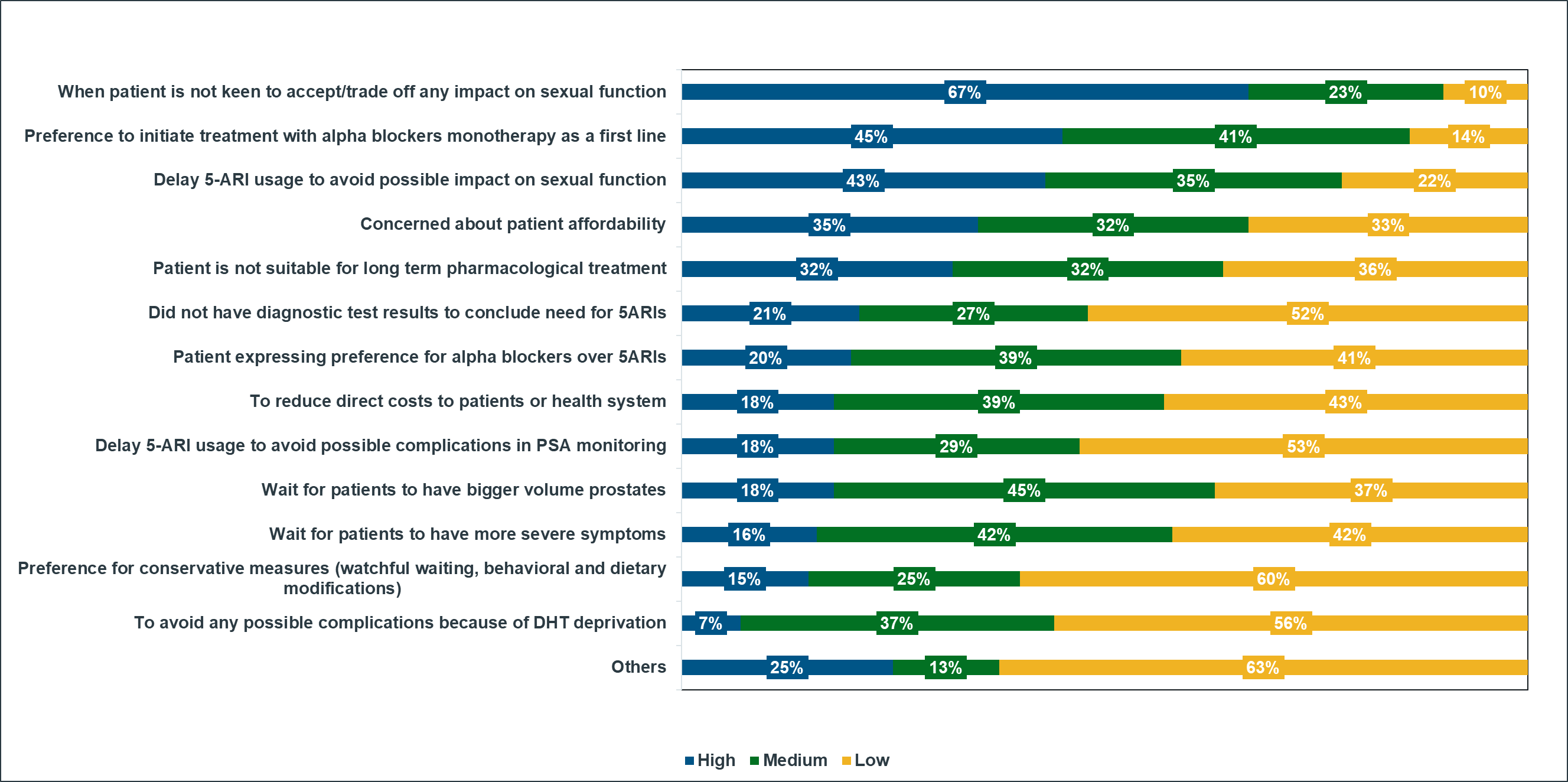
***

c)

***
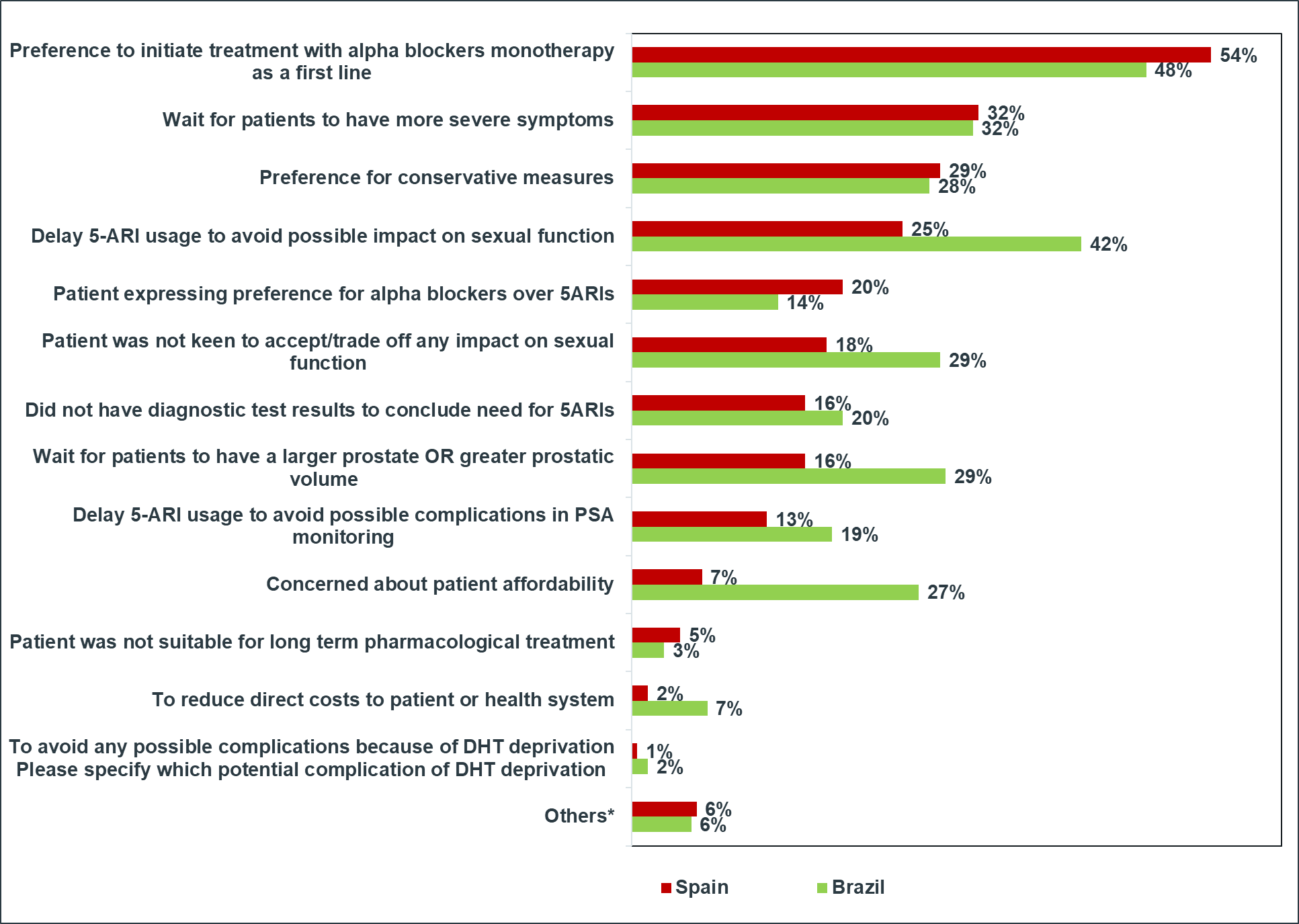
***

5ARI, 5-alpha-reductase inhibitor; BPH, benign prostatic hyperplasia; PRF, patient record form.

**Supplementary Figure 5:** Patient involvement in decision-making: (a) percentage of patients that urologists involve in discussions about treatment options per questionnaires; (b) percentage of discussions with patients that lead to treatment changes per questionnaires; and (c) percentage of cases where treatment options are discussed and lead to changes in therapy per PRFs.

a)


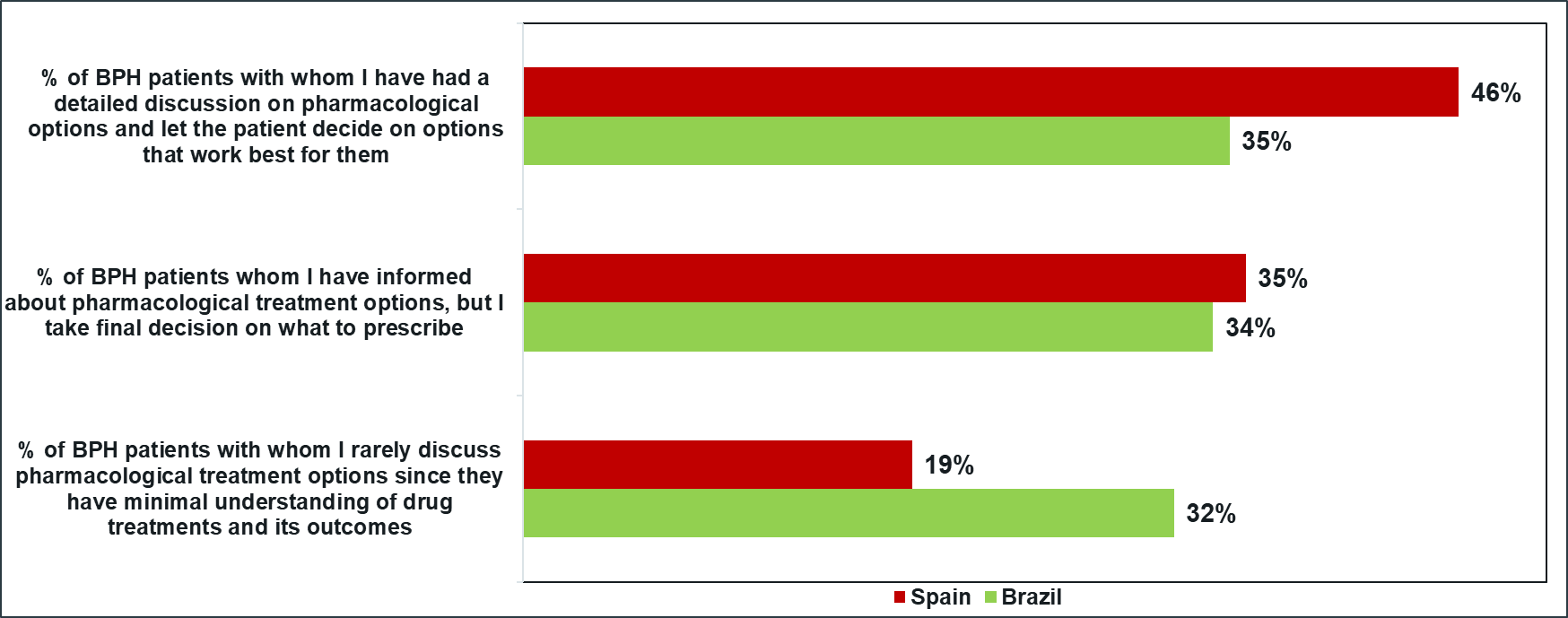


b)


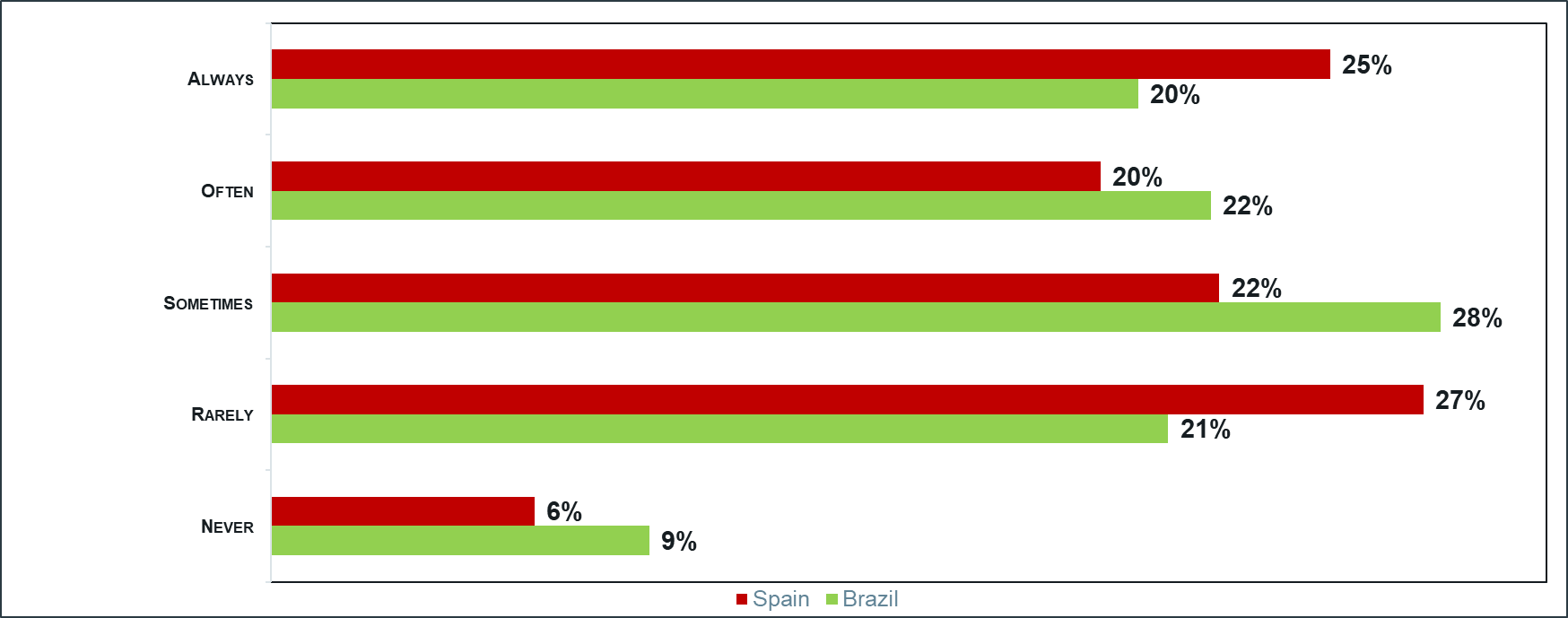


c)


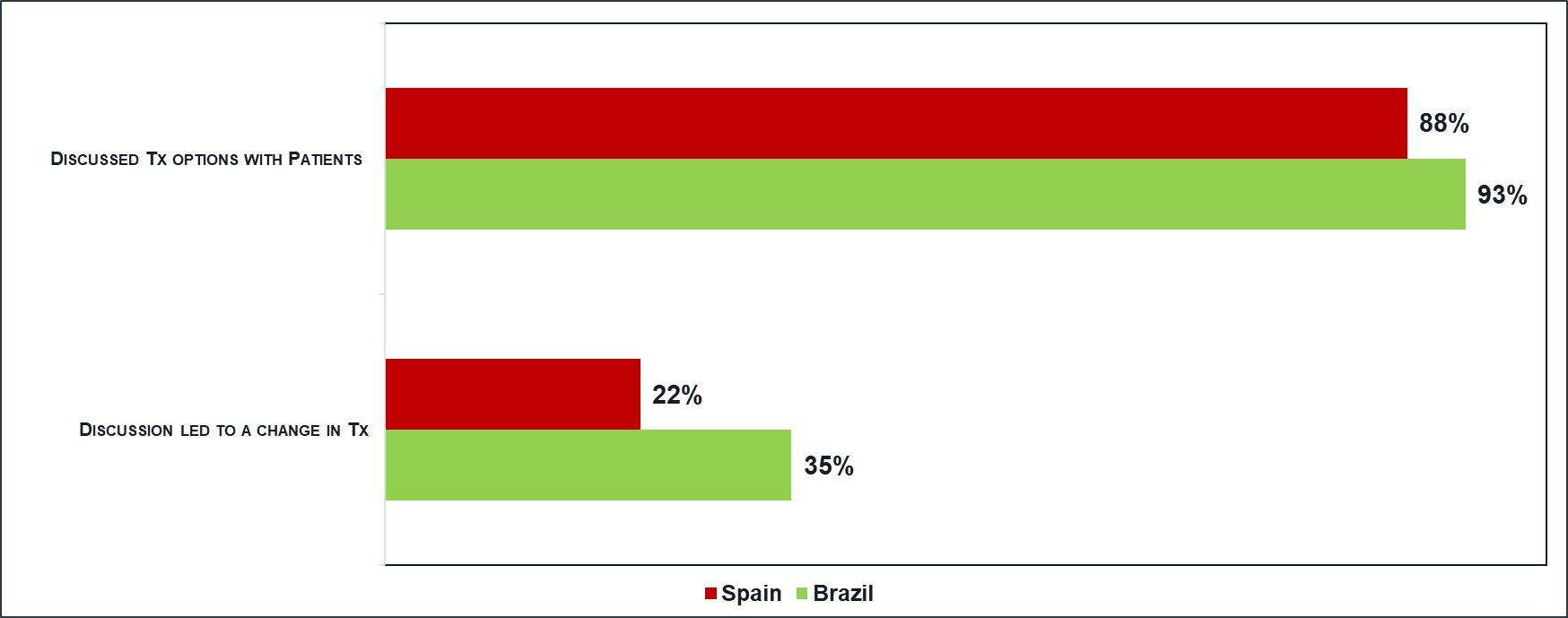


PRF, patient record form; Tx, treatment.

**Supplementary Table 1:** Characteristics of the urologists who participated in the survey.

| **Characteristic** | **Spain (*N* = 100)** | **Brazil (*N* = 100)** |
| --- | --- | --- |
| Mean experience, years | 16 | 14 |
| Practice type, n (%)  Public  Private | 87 (87)  13 (13) | 35 (35)  65 (65) |
| Average number of patients with LUTS/BPE  Managed in the last month  Currently receiving 5ARIs | 149  80 | 68  54 |

5ARI, 5-alpha-reductase inhibitor; BPE, benign prostatic enlargement; LUTS, lower urinary tract symptoms; SD, standard deviation.

**Supplementary Table 2:** Demographics of patients included in the PRFs.

| **Patient characteristic, n (%)** | **Spain**  **(*N* = 200)** | **Brazil**  **(*N* = 200)** |
| --- | --- | --- |
| Age, years  50–54  55–59  60–64  65–69  70–74  75–79  ≥80 | 9 (5)  41 (21)  51 (26)  28 (14)  41 (21)  19 (10)  11 (6) | 20 (10)  42 (21)  51 (26)  35 (18)  30 (15)  12 (6)  10 (5) |
| Time since diagnosis  <7 months  7–12 months  >12 months | 55 (28)  58 (29)  87 (44) | 52 (26)  45 (23)  103 (52) |
| Comorbidities  Hypertension  Diabetes  Dyslipidemia  Overweight/obesity  Cardiovascular disease  Sexual dysfunction  Non-prostatic malignancy  Neurological condition  No comorbidities | 133 (67)  92 (46)  91 (46)  73 (37)  55 (28)  53 (27)  4 (2)  4 (2)  18 (9) | 162 (81)  98 (49)  65 (33)  78 (39)  40 (20)  69 (35)  1 (0.5)  0  17 (9) |
| Type of sexual dysfunction  Erectile dysfunction  Ejaculatory dysfunction  Negative impact on libido/sexual desire  Negative impact on global sexual function | N=53  46 (87)  5 (9)  4 (8)  5 (9) | N=69  63 (91)  8 (12)  16 (23)  10 (15) |
| Risk categorization when 5ARI was initiated  At risk of progression  Not at risk of progression | *N* = 200  163 (82)  37 (19) | *N* = 200  176 (88)  24 (12) |
| Symptom severity based on IPSS  Severe  Moderate  Mild | *N* = 116  45 (39)  68 (59)  3 (3) | *N* = 51  13 (26)  35 (69)  3 (6) |
| Symptom severity per clinical assessment  Severe  Moderate  Mild | *N* = 81  30 (37)  49 (61)  2 (3) | *N* = 145  41 (28)  94 (65)  10 (7) |

*Each patient could have >1 comorbidity. Percentages may not total 100 due to rounding. Urologists were asked to provide details of 2 of most recent BPH patients aged 50 years or older, diagnosed with BPH and initiated with 5-ARIs treatment in the last one year (in monotherapy or combination with *α* blockers).

5ARI, 5-alpha-reductase inhibitor; IPSS, International Prostate Symptom Score; PRF, patient record form.

**Supplementary Table 3:** Reasons given for not assessing risk of progression in patients with LUTS/BPE.

| **Most common reason for not assessing risk of progression,**  **Urologist number (proportion)** | **Spain  (*N* = 100)** | **Brazil  (*N* = 100)** |
| --- | --- | --- |
| Short time at outpatient practice, *n* (%) | 55 (55) | 52 (52) |
| My priority is to tackle symptoms, not risk of progression, *n* (%) | 28 (28) | 22 (22) |
| I think risk of progression is overestimated in patients with BPH, *n* (%) | 25 (25) | 14 (14) |
| In my concept, risk of progression is not a decisive factor to choose treatment, *n* (%) | 16 (16) | 17 (17) |
| I must follow institutional/hospital guidance for treating patients and risk of progression is not formal part of the assessment, *n* (%) | 16 (16) | 9 (9) |
| I do not agree with clinical guidelines parameters and values that define risk  of progression, *n* (%) | 12 (12) | 5 (5) |
| Other, *n* (%) | 18 (18) | 21 (21) |

Percentages may not total 100 due to rounding.
BPE, benign prostatic enlargement; LUTS, lower urinary tract symptoms.
